# Unlinking the methylome pattern from nucleotide sequence, revealed by large-scale *in vivo* genome engineering and methylome editing in medaka fish

**DOI:** 10.1371/journal.pgen.1007123

**Published:** 2017-12-21

**Authors:** Napo K. M. Cheung, Ryohei Nakamura, Ayako Uno, Masahiko Kumagai, Hiroto S. Fukushima, Shinichi Morishita, Hiroyuki Takeda

**Affiliations:** 1 Department of Biological Sciences, Graduate School of Science, The University of Tokyo, Tokyo, Japan; 2 Department of Computational Biology and Medical Sciences, Graduate School of Frontier Sciences, The University of Tokyo, Tokyo, Japan; 3 CREST, Japan Science and Technology Agency, Kawaguchi, Japan; Friedrich Miescher Institute for Biomedical Research, SWITZERLAND

## Abstract

The heavily methylated vertebrate genomes are punctuated by stretches of poorly methylated DNA sequences that usually mark gene regulatory regions. It is known that the methylation state of these regions confers transcriptional control over their associated genes. Given its governance on the transcriptome, cellular functions and identity, genome-wide DNA methylation pattern is tightly regulated and evidently predefined. However, how is the methylation pattern determined *in vivo* remains enigmatic. Based on *in silico* and *in vitro* evidence, recent studies proposed that the regional hypomethylated state is primarily determined by local DNA sequence, e.g., high CpG density and presence of specific transcription factor binding sites. Nonetheless, the dependency of DNA methylation on nucleotide sequence has not been carefully validated in vertebrates *in vivo*. Herein, with the use of medaka (*Oryzias latipes*) as a model, the sequence dependency of DNA methylation was intensively tested *in vivo*. Our statistical modeling confirmed the strong statistical association between nucleotide sequence pattern and methylation state in the medaka genome. However, by manipulating the methylation state of a number of genomic sequences and reintegrating them into medaka embryos, we demonstrated that artificially conferred DNA methylation states were predominantly and robustly maintained *in vivo*, regardless of their sequences and endogenous states. This feature was also observed in the medaka transgene that had passed across generations. Thus, despite the observed statistical association, nucleotide sequence was unable to autonomously determine its own methylation state in medaka *in vivo*. Our results apparently argue against the notion of the governance on the DNA methylation by nucleotide sequence, but instead suggest the involvement of other epigenetic factors in defining and maintaining the DNA methylation landscape. Further investigation in other vertebrate models *in vivo* will be needed for the generalization of our observations made in medaka.

## Author summary

The genomes of vertebrate animals are naturally and extensively modified by methylation. The DNA methylation is essential to normal functions of cells, hence the whole animal, since it governs gene expression. Defects in the establishment and maintenance of proper methylation pattern are commonly associated with various developmental abnormalities and diseases. How exactly is the normal pattern defined in vertebrate animals is not fully understood, but recent researches with computational analyses and cultured cells suggested that DNA sequence is a primary determinant of the methylation pattern. This study encompasses the first experiments that rigorously test this notion in whole animal (medaka fish). In statistical sense, we observed the very strong correlation between DNA sequence and methylation state. However, by introducing unmethylated and artificially methylated native genomic DNA sequences into the genome, we demonstrated that the artificially conferred methylation states were robustly maintained in the animal, independent of the sequence and native state. Our results thus demonstrate that genome-wide DNA methylation pattern is not autonomously determined by the DNA sequence, which underpins the vital role of DNA methylation pattern as a core epigenetic element.

## Introduction

DNA methylation is central to the epigenetic control of transcription in vertebrates and is essential for cell differentiation and embryonic development [1–3]. While the cytosines in cytosine-guanine (CpG) dinucleotides are extensively methylated throughout vertebrate genomes, unmethylated CpGs are commonly found clustered at high density inside gene regulatory elements, such as promoters and enhancers. Previous studies have revealed that the methylation state of regulatory regions governs the expression of their associated genes [4,5]. Furthermore, aberrant changes in the methylation state can lead to deregulated transcription, resulting in cellular dysfunction, diseases and developmental abnormality [6,7].

Given its direct governance on transcription, the methylation landscape needs to be precisely specified and modulated. The DNA methylation pattern is established and maintained through highly dynamic biological processes, in which the methylome undergoes substantial, yet precise, changes. For instance, differentiating cells faithfully acquire specific methylation landscapes that are unique to their committed cell types [8–10]. Remarkably, in human and mice, the DNA methylome is extensively erased [11,12] and fully reconstituted during gametogenesis and early embryonic development [13–15]. These facts suggest that the methylation landscape is pre-defined by genetic information. Thus, deciphering how the methylation pattern is encoded is a prerequisite for understanding of differentiation processes and the pathogenesis of various diseases [6,16–18]. However, by what means the methylation pattern is defined *in vivo* remains enigmatic.

Researches for the past decade proposed that DNA methylation pattern depends on local sequence context. In particular, *in silico* analyses asserted that there is the strong statistical association between sequence variants and differential DNA methylation states in vertebrates, from fish [19] to human [20]. A number of recent *in vitro* studies using cultured cells further demonstrated that high CpG density or the presence of specific DNA sequences that contain transcription-factor binding sites is capable of autonomously determining local hypomethylation in the globally methylated genome [21–24]. These recent *in silico* and *in vitro* reports support the notion that DNA methylation pattern is primarily determined by local sequence context [21]. However, the anticipated sequence-dependency of DNA methylation is in contradiction to the pioneer *in vitro* experiments in early 80’S [25–27], in which the methylation status of exogenous DNAs (either artificially CpG-methylated or completely unmethylated) was found maintained with certain fidelity for many cell generations upon stable genome integration. Given these opposing results, the sequence-dependency of DNA methylome seems less concrete than recently anticipated.

Importantly, the above ideas have never been well demonstrated nor rigorously tested *in vivo*. In this respect, the report by Long *et al*. [28] provided valuable insights by studying the DNA methylation state of the 42-Mbp fragment of human chromosome 21 in the Tc1 trans-chromosomic mice, as well as the mouse genome loci-containing transgene constructs that were artificially transposed into the zebrafish genome. Their results suggested the existence of sequence-dependent DNA methylation *in vivo*, but their analyses only focused on non-native sequences (i.e. examining human genomic sequence in mouse, or mouse genomic sequence in zebrafish). Likewise, Li *et al*. [29] examined the methylation status of a transgene across three generations in rat and found the stable acquisition and inheritance of DNA methylation pattern, but the transgene examined was composed of a mouse promoter and human gene. Thus, it is difficult to draw a general conclusion with these studies on the causal relationship between DNA sequence and methylation in native context *in vivo*.

Herein, we report the first experiments that rigorously tested the governance of DNA methylation state by nucleotide sequence *in vivo*. The small laboratory fish, medaka (*Oryzias latipes*), was chosen as an experimental model for their relatively small genome size (approx. 700 Mbp), short generation time (2.5 to 3 months), ease of *in vivo* genetic manipulation, oviparity, in addition to their capability of producing 10–20 fertilized embryos per pair on daily basis [30,31]. Importantly, the medaka has polymorphic inbred lines from two geographically separated subpopulations living in the northern and southern part of Japan (2.5–3% SNP rate, for review, see [32]), and their genomes and methylomes were already decoded [19,33,34]. Although vertebrates could have variable DNA methylation dynamics, particularly during early embryonic development (e.g., the genome-wide methylation erasure immediately after fertilization is highly extensive in human and mice [11,12], but very subtle or virtually absent from sheep [35], medaka [36] and zebrafish [37]), the ultimate zygotic DNA methylation landscape is highly conserved from fish to mammals [37–39]. In addition, since an extensively methylated genome is believed to be prerequisite for the onset of vertebrate evolution [40–42], the molecular mechanisms and logic underlying the patterning of DNA methylome are likely conserved among vertebrates. Hence, observations made on medaka can potentially shed light on the postulated, yet unproven, link between genomic sequences and DNA methylation in vertebrates. Contrary to expectation, our results suggest that nucleotide sequence, by itself, cannot dictate its own methylation state *in vivo*, which argues against the prevailing view of DNA methylation in vertebrates.

## Results

### Hypomethylated and hypermethylated domains exhibit distinct sequence patterns

**Fig 1:**
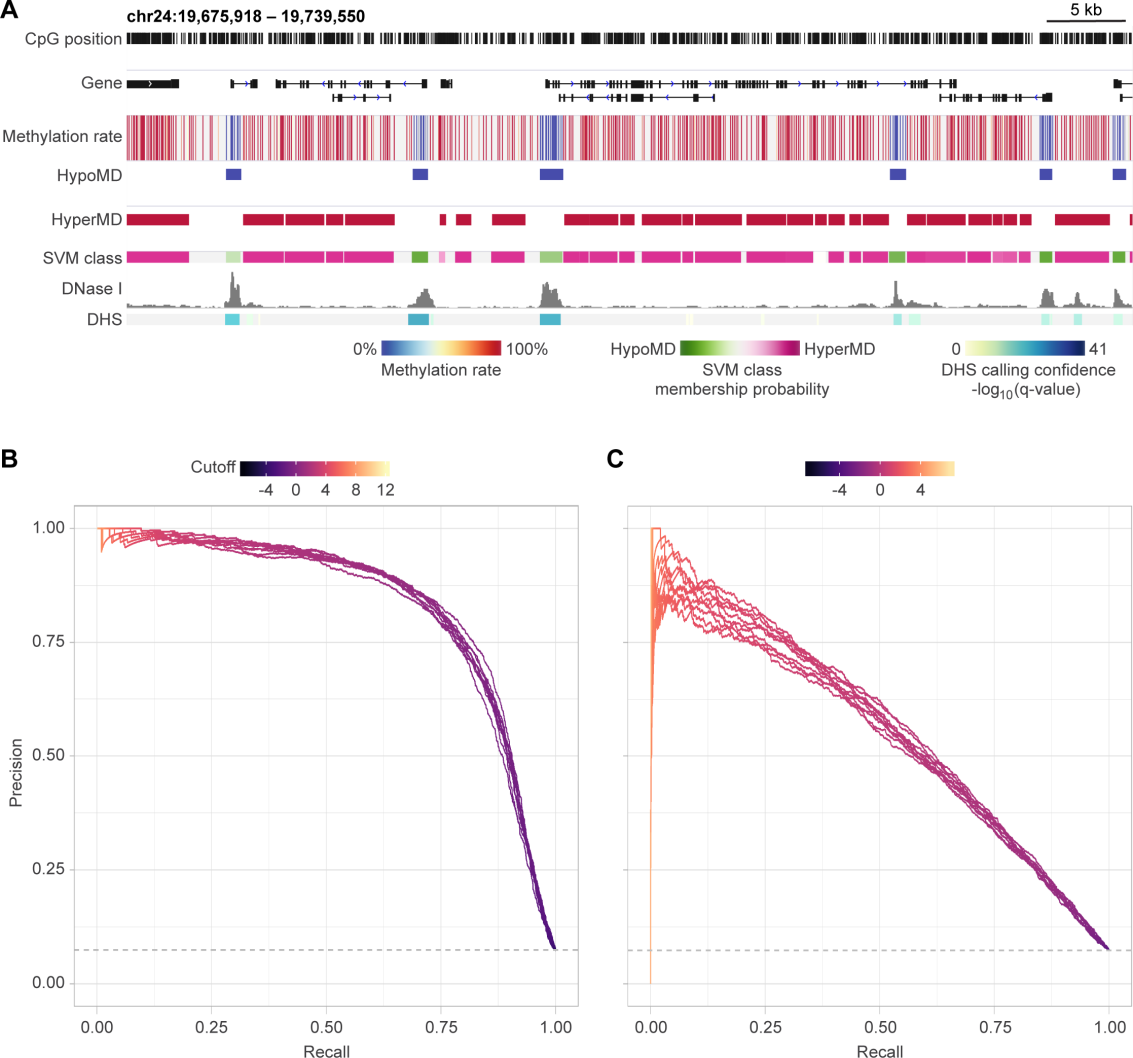
Strong statistical association between methylation state and genomic sequence in medaka. (A) Genome browser view of a representative locus (approx. 62 kb) in the HdrR medaka genome showing CpG methylation rate, the called HypoMDs and HyperMDs, the SVM classification results, as well as DNase I hypersensitivity and the called peaks (i.e. DNase I hypersensitive sites, “DHS”). (B & C) Precision-recall curves of the kmer-SVM models trained for binary classification of HypoMDs and HyperMDs (B) without- or (C) with- CpG-masking. HypoMD and HyperMD sequences were assigned to positive and negative classes, respectively. Solid, colored lines are individual precision-recall curves derived from 10-fold cross-validation. The colors represent the cut-off values for binary classification/prediction of the testing pool in each rounds of cross-validation. Area-under-curve (AUC): (B) minimum = 0.83, maximum = 0.84; (C) minimum = 0.53, maximum = 0.56. Random classifier is represented by horizontal dashes at the bottom of both panels and has an AUC of 0.08.

Statistical association between medaka genomic sequences and local methylation states was modelled using support vector machine (kmer-SVM [43]). Hypomethylated and hypermethylated genomic loci (a.k.a. hypomethylated domains, “HypoMDs”, and hypermethylated domains, “HyperMDs”, respectively) at the blastula stage (Stage 11 according to Iwamatsu [44]) were identified using the same criteria as described by Nakamura *et al*. [45] (see also Fig 1A). While HypoMDs and HyperMDs are not readily discernible in terms of length and GC composition (S1 Fig: panel A & B), they bear conspicuous difference in their sequence pattern, allowing robust *in silico* classification and accurate prediction of the methylation states by the SVMs based solely on nucleotide sequence (Fig 1B: area under precision-recall curve ≥ 0.83, versus 0.08 from the random classifier). Consistent with the fact that the median CpG density in HypoMDs is higher than that in HyperMDs (S1 Fig: panel C), sequence pattern enriched in HypoMDs display higher frequency of CpG (S1 Table: left columns). Furthermore, CpG-masking prior to the training of SVM could still result in models with modest classification performance (Fig 1C: area under precision-recall curve ≥ 0.53), suggesting that specific, CpG-free DNA motifs are also differentially enriched in HypoMDs and HyperMDs (S1 Table: columns on the right). All these reinforce the notion that, similar to other vertebrates, there is the strong statistical association between genomic DNA sequences and their methylation states in medaka.

### Local methylation state appears to be independent of nucleotide sequence for generations

**Fig 2:**
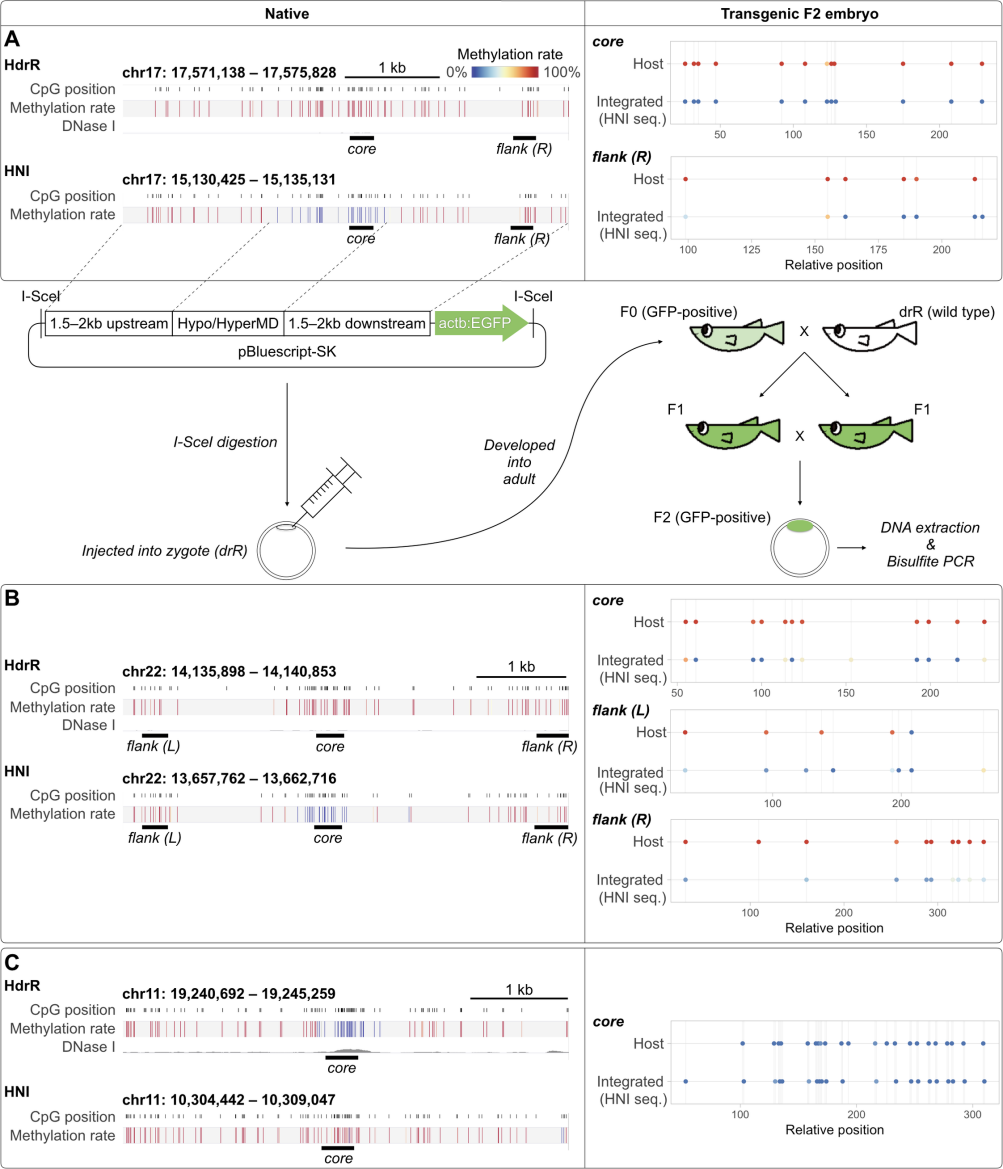
Stably integrated transgenes failed to recapitulate their endogenous methylation states in F2 transgenic embryos. (Left) Native methylation states, at blastula stage, of the homologous genome loci that are differentially methylated between HdrR and HNI medaka strains. The HNI loci, along with their 1.5–2 kb flanking hypermethylated regions (i.e. the whole region in display) were cloned and integrated into drR strain as transgenes. (A & B) Two loci that are hypermethylated in HdrR, but hypomethylated in HNI. (C) A locus that is hypomethylated in HdrR, but hypermethylated in HNI. (Right) Bisulfite PCR sequencing results showing the methylation state of the integrated HNI sequence in the F2 transgenic blastula drR embryos. “core” and “flank (L) / flank (R)” correspond to the endogenously differentially methylated regions and the flanking regions, respectively, shown on the left. The methylation state of the homologous genome regions in the transgenic drR embryos (“Host”) were also shown as reference. Note that the sampled regions (“core”, “flank (L)”, and “flank (R)”) inside the integrated HNI sequences were all poorly methylated regardless of their native states. Mean coverage = 11×.

To test the dependency between genomic sequences and their methylation state *in vivo*, we generated transgenic fish that ectopically carry full-length HypoMD or HyperMD, along with their 1.5 to 2-kb up- and down-stream sequences. To distinguish the endogenous and the ectopic copies of the assayed sequences, we specifically selected HypoMD and HyperMD that are differentially methylated in two closely related, inbred strains of medaka: HdrR and HNI [32], i.e. being a HyperMD in HdrR but exists as HypoMD in HNI, or *vice versa* (Fig 2). The differential states of these homologous sequences in the two strains were presumably due to minor variation in their nucleotide sequences [19]. These transgenic fish helped reveal not only if the differential methylation state is genuinely due to sequence polymorphisms, but also if genomic sequence at ectopic loci could stably recapitulate its endogenous state over a substantial timeframe and across generations (i.e. > 6 months, for the collection of F2 transgenic embryos).

Three transgenic lines were examined, in which DNA sequences from HNI (either endogenously HyperMD or HypoMD) were inserted into the host drR strain (outbred, parental strain of HdrR) (see Fig 2 for schematic illustration). Host drR and inserted HNI sequences were easily discriminated by SNPs. In concordance with the notion that nucleotide sequence can autonomously determine its own methylation state, the integrated full-length HypoMDs were completely unmethylated in the F2 transgenic blastula embryos (Fig 2A & 2B: “core”). However, on the other hand, the integrated full-length HyperMD (Fig 2C: “core”) were also found poorly methylated in the transgenic embryos, which is in stark contrast to its native hypermethylated state. Moreover, while all flanking sequences tested are endogenously hypermethylated in both strains, they were poorly methylated ectopically (Fig 2A–2C: “flank (L)” and “flank (R)”). In fact, substantial *de novo* methylation was not evident throughout all three integrated sequences, regardless of inside HypoMD, HyperMD, or their flanking regions. Since the transgene constructs were initially propagated in *E*. *coli* as bacterial plasmids and were thus completely devoid of CpG methylation prior to transgenesis, these observations suggested that the initial absence of CpG methylation on the transgenes was faithfully maintained regardless of their sequence and respective endogenous methylation states for at least 6 months and across 3 animal generations. This indicates that these assayed genomic sequences (1) do not carry methylation determination information and/or (2) randomly integrated into loci (e.g., inside or in close proximity to expression cassettes) that were under strong influence of preexisting epigenetic factors.

### Methylation states are not autonomously determined by nucleotide sequence at ectopic genomic positions

**Fig 3:**
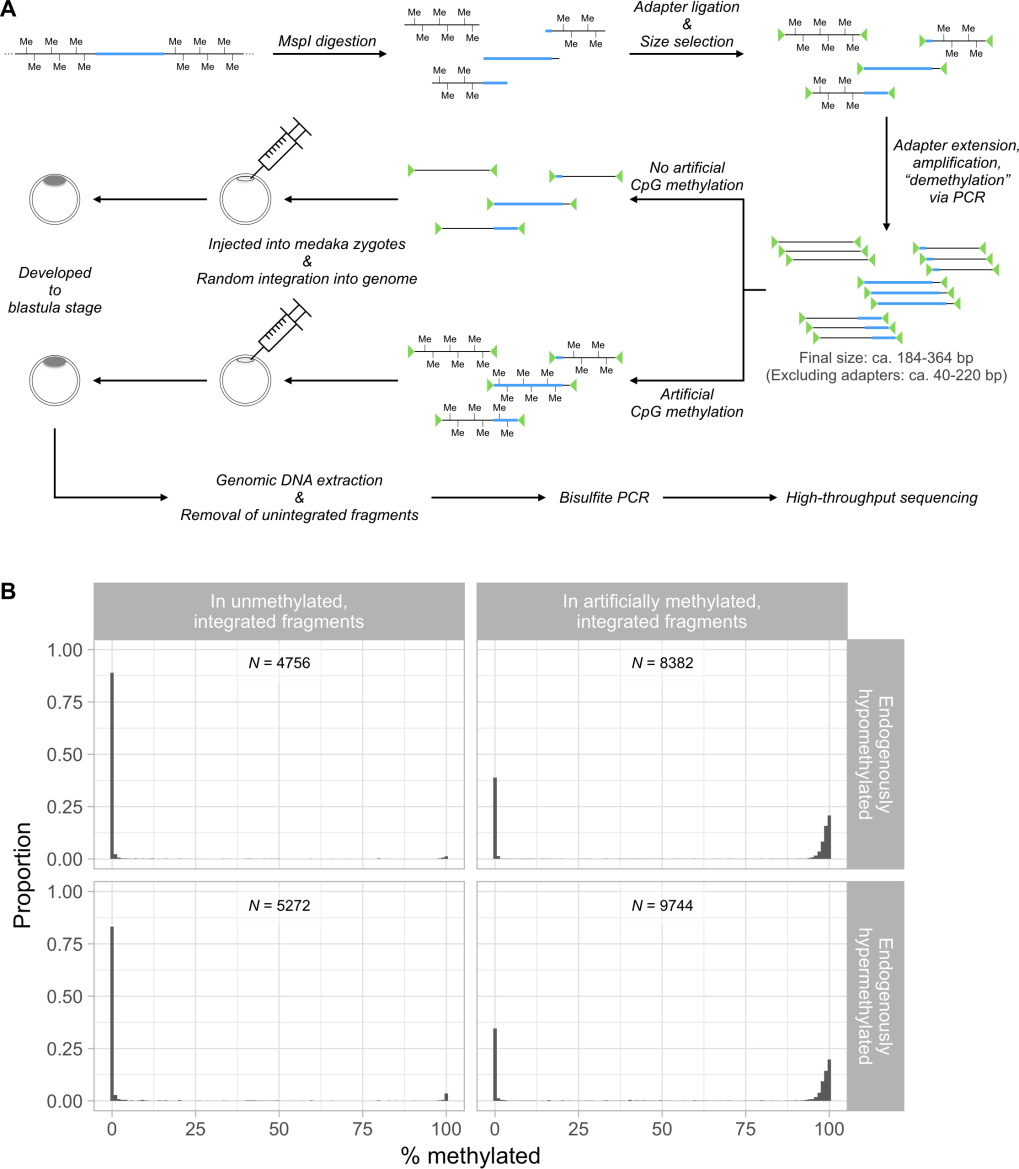
Randomly integrated genomic fragments could not autonomously determine their methylation state. (A) Schematic diagram illustrating the capturing and processing of genomic fragments for the interrogation of their autonomy in methylation state determination. The blue segment represents genomic region that is endogenously hypomethylated. (B) Distributions of the methylation rates of CpGs on the integrated genomic fragments, (left) without- or (right) with- artificial methylation prior to injection. The distributions were displayed separately for CpGs that are endogenously (upper) hypomethylated and (lower) hypermethylated. Bin width = 1%. Note that the histograms in the upper panels (i.e. CpGs that are endogenously hypomethylated) strongly resemble those in the lower panels (i.e. CpGs that are endogenously hypermethylated). “*N*” denotes the number of CpGs in the corresponding histograms. Mean coverage = (left) 292× and (right) 208×.

Given the above unexpected observations, a substantial number of genomic fragments of medaka was interrogated to comprehensively test the general presumption that genomic sequences can genuinely determine their own DNA methylation state *in vivo*. Medaka genomic DNA was digested and enriched for CpG-containing fragments (approx. 40–220 bp; extended to approx. 184–364 bp with adapters) using a library preparation method akin to that was designed for reduced representation bisulfite sequencing (RRBS) [46]. The PCR-amplified (hence, unmethylated) fragments were labeled (methylation at the N6 position of adenine in the Dam sites, 5’-GATC-3’, of the adapters), followed by (or without) artificial CpG methylation *in vitro*, then introduced into medaka zygotes at the one-cell stage, and allowed for highly efficient I-SceI-mediated random genome integration (see Fig 3A for graphical procedures). According to Thermes *et al*. [47], the integration event was expected to occur at the one-cell stage, i.e. immediately after injection. At the blastula stage (2000 to 4000 cells per embryo), after the removal of unintegrated fragments by size-selection and DpnI-digestion (S2 Fig: panel A), the methylation state of the integrated fragments was determined via bisulfite PCR and high-throughput sequencing. The assayed integrated fragments encompassed nearly the entire range of GC content and CpG density of HypoMDs and HyperMDs (S3 Fig vs S1 Fig). Approximately equal number of CpGs from HypoMDs and HyperMDs were assayed (S4 Fig: top vs bottom panels).

In spite of the strong statistical association between nucleotide sequence and methylation states, the integrated genomic fragments failed to recapitulate their endogenous methylation state at ectopic locations. The methylation rate at endogenous loci and that at ectopically integrated locations showed essentially zero statistical correlation: Spearman’s ρ ≤ 0.08, Kendall’s τ ≤ 0.07 (see also S5 Fig for the biplots). Without prior artificial methylation, CpGs on the integrated fragments were almost entirely unmethylated regardless of their endogenous states (Fig 3B: upper-left vs lower-left panel). The lack of sequence dependency was further illustrated by a drastically different ectopic methylation pattern when the genomic fragments were artificially methylated prior to injection and genome integration (Fig 3B: left panels vs right panels). The sharp contrast in the ectopic methylation patterns suggested that nucleotide sequence does not carry adequate information for its own methylation state, or the integrated fragments could escape *de novo* DNA methylation (which occurs at some point between 64-cell stage and blastula stage [36]) and any expected sequence-dependent demethylation in early medaka embryos.

The artificially methylated, integrated fragments contained a substantial number of unmethylated CpGs when examined at the blastula stage (Fig 3B: upper- and lower-right panels). These unmethylated CpGs were unlikely due to incomplete artificial methylation prior to injection for the following reasons. The methylase (CpG DNA methyltransferases M.SssI) used is known to completely methylate CpGs in all sequence context [48]. This was routinely achievable by our optimized reaction regimen (see S6 Fig for examples using bacterial genomic DNA and vector library that have higher CpG frequencies per unit weight of DNA than the medaka genome). The observed unmethylated CpGs could be caused by demethylation in the injected embryos. However, such demethylation could not be directly inferred as recapitulation of the endogenous methylation state, since there was essentially zero correlation between the endogenous and ectopic states (Fig 3B: upper-right vs lower-right panel; see also panel B in S5 Fig). In addition, the observed loss of premethylated state was unrelated to the endogenous chromatin accessibility (hence, potential binding of- or recognition by- transcription factors), as CpGs originated from heterochromatin and euchromatin were equally susceptible to the loss of methylation (right panels of S7 Fig; note the peaks at 0% methylation rate in the histograms along Y-axes). We also compared the nucleotide sequences (10 bp from both up- and down-stream) encompassing CpGs that were demethylated to those that were maintained as hypermethylated using kmer-SVM with the same parameters as above. However, the resultant SVMs were highly imprecise and insensitive (S8 Fig: area under precision-recall curve ≤ 0.47, versus 0.43 from random classifier). Moreover, the overall ectopic methylation states, as well as the demethylation, of the integrated fragments do not correlate with their size or CpG density (S9 Fig). Together, we concluded that the observed demethylated state was not related to intrinsic sequence features of the genomic fragments.

Given that the injected genomic fragments were (1) only partial fragments of HypoMDs or HyperMDs and may lack the presumed sequence features that are required for autonomous determination of methylation state, and (2) integrated into random genomic positions where they might be influenced by local chromatin state, we speculated that the observed demethylation might be due, at least in part, to the local epigenetic state of the integrated loci (i.e. position effect; E.g., integrated into preexisting HypoMDs or somewhere under the influence of *trans*-acting hypomethylation determining elements, hence rendered hypomethylated). Subsequent experiments were thus conducted at pre-specified genomic loci to control for the possible position effect.

### Methylation state is maintained independent of sequence and position context

**Fig 4:**
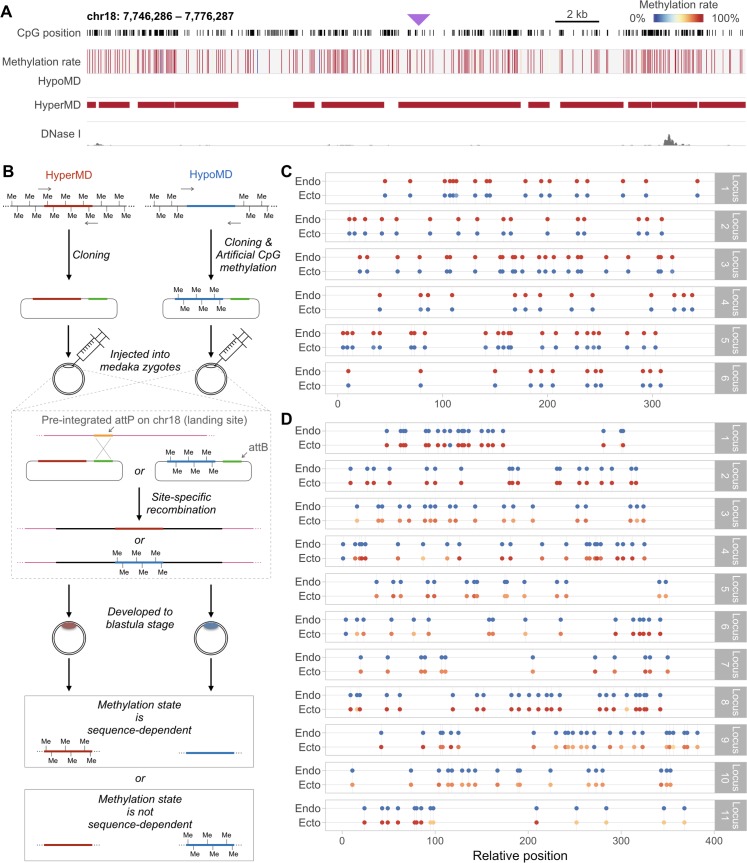
Artificially conferred methylation states were maintained by full-length HyperMD and HypoMD sequences after being inserted into a gene desert. (A) Genome browser view of the methylation states (as in HdrR strain) of the genomic locus that contains the landing site for PhiC31-mediated site-specific recombination (approximate location is denoted by the purple triangle). (B) Schematic diagram illustrating the irreversible, site-specific integration of the subcloned, unmethylated HyperMDs and pre-methylated HypoMDs via PhiC31-integrase-mediated site-specific recombination and the expected outcomes depending on whether the integrated sequences can autonomously determine their own methylation state. (C) Methylation state of six HyperMDs at their endogenous loci (with reference to published whole genome bisulfite sequencing dataset; see also Materials and Methods) and at the ectopic location after being cloned and integrated into genome via PhiC31-mediated site-specific recombination. Note that all of the integrated sequences failed to recapitulate their endogenous hypermethylated state. (D) Methylation state of eleven HypoMDs at their endogenous loci and at the ectopic location after being cloned, artificially methylated, then integrated into genome via site-specific recombination. All of the pre-methylated, integrated sequences failed to recapitulate their endogenous hypomethylated state. Complete loss of methylation was only observed in very small number of CpGs in four of the examined sequences: the 9^th^ CpG of Locus 1, the 1^st^ CpGs of Locus 4, the 1^st^ CpG of Locus 6, and the 14^th^ CpG of Locus 9. Mean coverage = (C) 20× and (D) 15×.

In order to examine whether full-length HyperMDs and HypoMDs can autonomously determine their own methylation state at an inert genomic location, six unmethylated HyperMDs and eleven pre-methylated HypoMDs were injected into one-cell stage medaka embryos and integrated into the gene desert region presumably devoid of any possible influence of active regulatory elements (see also Fig 4A). The integration was achieved by the highly efficient, PhiC31 integrase-mediated site-specific integration in medaka and was expected to occur at the one-cell stage [49]. Methylation states of the integrated sequences were examined at the blastula stage. Autonomy in methylation state determination by the full-length, integrated sequences would manifest as remethylation of the unmethylated HyperMDs, as well as active or passive loss of the methyl groups on the premethylated HypoMDs, after genome integration (see also Fig 4B for illustration of the logic of the experiment).

In concordance with the above experiments, all of the unmethylated, integrated HyperMDs failed to acquire methylation (Fig 4C). Likewise, the pre-methylated, integrated HypoMDs remained hypermethylated (Fig 4D), with very limited number of CpG dinucleotides (i.e. only 4 out of the 202 CpGs inspected) having no methylation (Fig 4D: blue dots on the integrated, ectopic copies of HypoMDs/Loci 1, 4, 6, and 9). Since we were unable to determine the methylation state of these distinct CpGs in the premethylated plasmid library (as plasmid DNA converts very poorly in bisulfite reaction), it is possible that these CpGs were not fully methylated prior to injection. However, as aforementioned, the M.SssI methyltransferase used in the pretreatment has no known sequence specificity. The observed absence of methylation probably reflects highly localized loss of methyl groups on these specific CpGs. Collectively, the above results indicate that the overall, ectopically introduced nucleotide sequences were not perused and the artificially conferred methylation states (i.e. hypomethylation in the HyperMDs, and hypermethylation in the HypoMDs) were robustly maintained *in vivo*.

**Fig 5:**
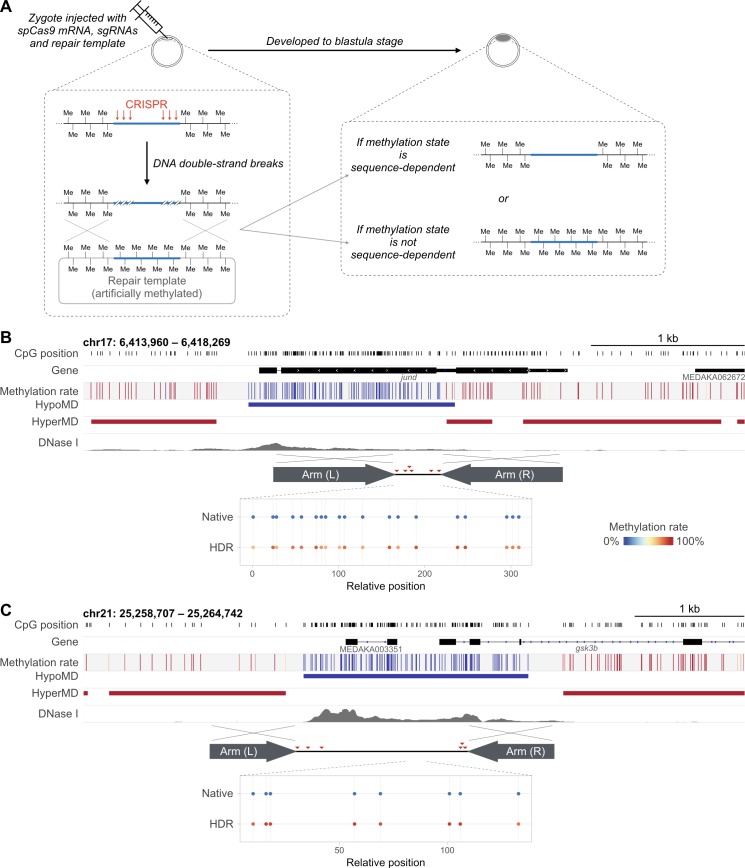
HypoMDs could not restore their native methylation state at their endogenous loci after “methylome editing”. (A) Schematic diagram illustrating the principle of the methylation state editing on the targeted HypoMDs via homology directed repair (HDR) and the use of artificially methylated repair template. HDR was triggered by CRISPR-Cas9 induced DNA double-strand breaks (DSBs) at the targeted loci. The repair template contained the subcloned HypoMD (with substitutions in the spCas9’s PAM sites, from 5’-NGG-3’ to 5’-NGC-3’) along with approximately 800 bp flanking regions that served as homology arms. Note that multiple DSBs were made using a cocktail of sgRNAs that guided spCas9 to six different positions along the targeted HypoMD to enhance DSB, hence HDR, rate. (B & C) Methylation state of two HypoMDs after *in vivo* methylome editing mediated by CRISPR-Cas9-induced homology-directed repair (HDR) and pre-methylated repair templates. The estimated methylation rates were normalized against the estimated editing rate (see Materials and Methods). Red triangles: binding positions of the sgRNAs.

Finally, we edited the methylation state *in situ* to exclude the risk of artifacts possibly incurred by ectopic genome locations. The methylation state of two HypoMDs were edited *in situ* via CRISPR-Cas9-triggered homology directed repair (HDR) and artificially methylated repair templates (see Fig 5A for illustration of concept behind the experiment). Consistent with the aforementioned observations, in spite of the original hypomethylated state, the loci were rendered largely hypermethylated in the edited blastula embryos (Fig 5B & 5C). Since the observed lack of restoration of native methylation state could be due to the seemingly limited time allowed for the recapitulation (from injection to sampling, i.e. from 1-cell stage to blastula: approx. 8 hrs, encompassing 11–12 rounds of cell divisions), we repeated the editing experiment and extended the endpoints to later developmental stages at 3- (Stage 31) and 7- (50% hatched and free-swimming; i.e. Stage 39) day-post-fertilization (i.e. day-post-injection). Yet, the edited alleles remained hypermethylated in the mid-/late-stage embryos (S10 Fig). Significant loss of methyl groups could only be observed on two distinct, adjacent CpGs in one of the two edited loci (S10 Fig, panel B: the 1^st^ and 2^nd^ CpG). Taken together, these observations indicate that genomic sequence and its methylation state were not coupled even at the endogenous position.

## Discussion

Although DNA methylation is the best characterized epigenetic signature [50], the molecular basis and logic of its establishment still remain elusive. Given that CpG dyads are predominantly methylated unless they are clustered at high density [51], it is generally presumed that hypermethylation is the default state of vertebrate genomes and specific regions (i.e. gene regulatory elements) are protected from *de novo* methylation, rendering them hypomethylated [21,24,52–55]. Intensive researches for the past decade have demonstrated that the protection on the genomic loci is possibly mediated by nucleosome positioning [56–58] and/or the recruitment of a myriad of proteins [12,59–63] which eventually block off local access of DNA methyltransferases or remove methylation on cytosines in vicinity through oxidation and thymine DNA glycosylase (TDG)-mediated base excision repair. However, little is known about how are these factors specifically predisposed on the preselected loci.

As aforementioned, recent *in vitro* studies demonstrated that nucleotide sequence features (especially high CpG density and the presence of certain transcription factor binding sites) autonomously determined the local hypomethylated state [21–24,64,65]. However, this preposition has never been rigorously verified *in vivo*, presumably due to the fact that interrogation of genomic sequences at genome-wide scale requires large number of subject animals, which is prohibitive with classical mammalian models (e.g., rodents). With the use of medaka as an alternative vertebrate model, our results definitively showed that there is no immediate connection between DNA methylation state and underlying nucleotide sequence *in vivo*, in spite of their strong statistical association. By manipulating and controlling the methylation state of genomic sequences prior to reintegration into the genome, we demonstrated that the artificially established methylation states were predominantly maintained in medaka *in vivo*, independent of their nucleotide sequences and native methylation states. This also appears to be true for the transgene that passed across generations. Our results thus argue against not only the recently inferred determining role of DNA sequence on the methylation landscape, but also the longstanding belief that there is a default state (i.e. hypermethylated) for the vertebrate genomes.

In fact, the postulated strict sequence-dependency seems paradoxical to the concept of epigenetics itself. There are accumulating reports for the last two decades that DNA methylation could be perturbed by transient physiological stress or chemical exposure. More importantly, the perturbed states could be highly persistent and inheritable, while the underlying genomic sequence remains unchanged [66–68]. These observations highlighted that DNA methylation pattern is not directly coupled with the underlying nucleotide sequence *in vivo*, in spite of what has been recently shown *in silico* and *in vitro*.

However, our results do not rule out the existence of highly confined, local sequence-dependent DNA methylation. As proposed by Richards [69], the sequence-dependency of epigenetic signatures may vary with actual sequence-context, i.e. some nucleotide sequences may favor or even fully mandate certain methylation state, while others may be completely independent of DNA methylation. Although the artificially established hypermethylated state of HypoMD sequences examined in this study was mostly maintained after genome integration, we observed spontaneous, complete loss of methyl groups on some CpGs in the eleven pre-methylated HypoMDs, as well as within one of the *in situ* edited loci. This suggests the presence of local sequence elements that facilitate demethylation on specific CpGs, although their effect was spatially confined. As previously demonstrated *in vitro*, some DNA motifs, in particular several transcription factor binding sites (reviewed by Blattler and Farnham [70]), are indeed instructive to DNA methylation and may account for the change of methylation state in specific loci upon differentiation [18,71]. Importantly, their effect was also demonstrated to be limited to no more than a few tens of base pairs up- and down-stream [22,23]. It is thus likely that the restricted governing range (< 100 bp) of these DNA sequences is insufficient to account for the span of HypoMDs (median length > 1 kb).

The apparent lack of sequence dependency can be explained by the involvement of epigenetic factors in DNA methylation *in vivo*, as suggested by Kaminsky *et al*. [20]. Genomic fragments tested in the present study and previous works were all purified prior to reintegration into the genome, hence lacked any associated factor(s) that can modulate DNA methylation. Future experiments will need to address the presumed methylation determining factor(s), their deposition onto specific locations of the genome, and their inheritance across cell division and animal generations. The strong link between DNA methylation, nucleosome position and histone modifications [72–74] could provide a hint for further investigation.

In summary, with the use of medaka as a vertebrate model, our data presented herein oppose the recent proposition that the genome-wide DNA methylation pattern in vertebrates is primarily and autonomously designated by the underlying genomic sequence *in vivo*, but instead provide insights into potential involvement of other epigenetic factor(s) in defining the DNA methylation landscape. Our results demonstrate that the DNA methylation landscape and genomic sequence are not directly coupled, which underpin the widely-observed plasticity of DNA methylation along differentiation, as well as the transgenerational inheritance of perturbed DNA methylation *in vivo*. However, it is worth noting that vertebrate species could have variable methylation dynamics of DNA methylation during development and growth, especially during early embryonic stages, although underlying molecular mechanisms are probably conserved. This is true even within the same clade of vertebrate species, such as mammals [35]. Further investigation in other vertebrate models will definitely be needed before generalization of our observations made on medaka.

## Materials and methods

### Ethics statement

The culture and handling of medaka and their embryos followed the protocols and guidelines published in "Medaka: Biology, Management, and Experimental Protocols" (ISBN: 9780813808710). Experiments were conducted with the permission of Life Science Research Ethics and Safety committee of the University of Tokyo (Permission number: 14–05).

### HypoMD and HyperMD calling

Published whole genome bisulfite sequencing reads of medaka blastula embryos [75] were fetched from the Data Bank of Japan (accession number: SRX149583). Individual reads were trimmed to remove primers, adapters, and low quality basecalls (Phred score ≤ 3) using BBDuk from the BBTools ver. 35.85 [76]. Trimmed reads were mapped to the latest (as of the time of this writing) medaka genome assembly ver. 2.2.4 [34,77] (all genome coordinates reported herein refer to this assembly version) using bwa-meth ver. 0.2.0 [78]. Methylation rates of the mapped CpG dyads were then extracted using MethylDackel ver. 0.2.1 [79] with the default quality filters of MAPQ score ≥ 10 and Phred score ≥ 5. Only those CpG dinucleotides with coverage of ≥ 5× were considered as valid calls [75] and the final mean coverage after filtering was 8×. The same filtering criteria were also applied to all experiments throughout this study, wherever they are applicable. And, unless otherwise specified, the endogenous methylation states of sequences assayed in this study were directly extracted from this mapped, filtered dataset.

HypoMDs calling followed the same definition as previously published [19,45]. Specifically, any stretch of ten or more hypomethylated (methylation rate < 40%) CpGs with no more than four interleaving non-hypomethylated (methylation rate ≥ 40%) or undetermined (unsampled, unmappable or low coverage) dyads were called as HypoMD. HyperMDs were analogously defined as any stretch of at least ten hypermethylated (methylation rate > 60%) CpG dyads containing no more than four interleaving non-hypermethylated (methylation rate ≤ 60%) or undetermined CpGs.

### Supervised classification of HypoMD and HyperMD using support vector machine

To elucidate whether HypoMDs and HyperMDs contains distinct sequence features, genomic sequences of all called HypoMDs (*N* = 18435) and HyperMDs (*N* = 231516) were subjected to supervised classification using kmer-SVM (support vector machine with string-, i.e. nucleotide sequences-, based spectrum kernel) [43]. The default, recommended parameters and *k* = 6 (i.e. 6-mer) were used. Proportionally higher weights were assigned to HypoMDs (weight = 231516 / 18435 = 12.56) than HyperMDs (weight = 1) to offset the imbalanced sample sizes. Classification performance was gauged by 10-fold cross-validation and the area under precision-recall curves. Since HypoMDs have a higher average CpG density than HyperMDs (S1 Fig: panel C), CpG density might act as a confounding factor that outweighs and conceals non-CpG-containing sequence features. The impact of CpG density was hence controlled for by masking all CpG dinucleotides (i.e. from ‘CG’ to ‘NN’) and the SVM model was retraining using the same parameters as listed above.

### Generation of transgenic fish that carry HypoMD or HyperMD at ectopic genomic loci

Genomic regions that show contrasting methylation state between the HdrR and HNI strains were identified as described by Uno *et al*. [19]. HNI-specific HyperMD and HypoMDs, along with their 1.5 to 2-kb upstream and downstream sequences, were randomly selected and cloned using primers pairs F2-01 through F2-03 (see S2 Table for the oligo sequences). Medaka’s beta-actin promoter and EGFP coding sequences was amplified using primer sets F2-04 and F2-05, respectively. Amplified fragments were stitched together and cloned into pBlueScript-SK using In-Fushion assembly mix (Clonetech, Japan). The vectors were pre-treated with 5 units of I-SceI meganuclease in 20 μL of 1× I-SceI digestion buffer (New England Biolabs, USA) at room temperature for 1 hour and injected into medaka (drR strain) embryos at 1-cell stage following standard procedures [30]. Embryos that displayed stable, ubiquitously strong GFP fluorescence were raised and crossed with wild-type drR fish. GFP-positive F1 were inter-crossed to produce F2 generation. GFP-positive F2 embryos at blastula stage, i.e. Stage 11 by Iwamatsu [44], were sampled for genomic DNA extraction (see Method 1 in S1 Text). The purified genomic DNA was then bisulfite-converted using the MethylEasy Xceed Rapid DNA Bisulphite Modification Kit (Genetic Signatures, Australia) following manufacturer’s recommended procedures, except that DNA denaturation was carried out at 42°C for 20 mins. The stably integrated Hypo/HyperMDs and their flanking regions were PCR-amplified using BSP primers designed in MethPrimer [80] (primer set F2-06 through F2-11) and ExTaq polymerase (Takara Bio, Japan) under reaction conditions listed in Method 4 in S1 Text. BSP products were TA-cloned using TOPO TA Cloning Kit, Dual Promoter (Thermo Fisher Scientific, USA) and Sanger-sequenced (outsourced to FASMAC Co, Japan). Quality check and methylation rate quantification were carried out in QUMA [81] ver. 1.1.3 with default parameters.

### High-throughput transplantation of CpG-rich genomic loci

To test whether nucleotide sequences can autonomously determine their own methylation state *in vivo* at genome-wide scale, CpG-rich genomic fragments were captured and injected into medaka zygotes for random reintegration into the genome, then fished out to check for their methylation state. The capturing method was akin to those described for reduced representation bisulfite sequencing (RRBS). In fact, procedures up to the size selection of adaptor-ligated genomic fragments closely followed those optimized for RRBS [46]. The adaptor-ligated fragments were then enriched and amplified by extension PCR, which also introduced (from 5’ to 3’, in this order) I-SceI target sites, bisulfite PCR (BSP) primer binding sites (i.e. for primer F3-01F and F3-01R), and the Dam methylation site (5’-GATC-3’) to the products’ termini. The pool of amplified fragments was then Dam-methylated by incubating with Dam methylase (New England Biolabs) to facilitate downstream counter-selection of unintegrated fragments. Dam-methylated fragments were split into two equal halves with one half used directly for injection after purification and the other half subjected to artificial methylation using CpG methytransferase M.SssI (New England Biolabs) prior to injection. Detailed procedures are available as supplementary information (Method 2 & 3 in S1 Text).

Immediately prior to injection, the fragments (final concentration: 10 ng/μL) were pre-treated I-SceI meganuclease as above. Medaka zygotes were injected with Dam-methylated or Dam+CpG-methylated fragments at 1-cell stage. Around 500 embryos were injected with each pools of fragments and were allowed to develop to the blastula stage at 28°C. The embryos were visually inspected under dissecting microscope with dead or malformed embryos discarded. Ultimately, 496 (86%) and 433 (92%) embryos injected with Dam-methylated and Dam+CpG-methylated fragments, respectively, developed normally to the blastula stage, and from which genomic DNA with fragments integrated was extracted (Method 1 in S1 Text). While most of the unintegrated fragments were presumably removed using our optimized DNA extraction method that includes size selection by PEG precipitation, carryover was further minimized by incubating the extracted DNA with 2 μL of FastDigest DpnI (Thermo Fisher Scientific, USA) in a 20 μL of 1X NEB Buffer 2 (New England Biolabs) for a total of 72 hours at 37°C in an incubator. This was followed by routine phenol-chloroform extraction and isopropanol precipitation. The precipitated DNA was finally re-dissolved in 20 μL of freshly dispensed Milli-Q water (Merck Millipore, USA).

Efficient removal of unintegrated fragments was indicated by the parallel use of uninjected, spike-in control. Approximately twice the amount of the injection cocktail was spiked into the lysate of uninjected blastula embryos, which was then processed as described above. Relative quantity of library with or without integration was gauged by real-time PCR (THUNDERBIRD SYBR qPCR Master Mix, TOYOBO, Japan; in Agilent Stratagene Mx3000P, USA) using the library-specific primers F3-01F and F3-01R. In parallel, input DNA was also quantitated using primers F3-04F and F3-04R. Amplification plots were imported into qpcR v1.4.0 [82], where the relative quantities were determined after sigmoidal modeling (all adjusted R^2^ = 1.00).

The purified genomic DNA was then bisulfite-converted as above. Integrated fragments were enriched via PCR using primers F3-01F and F3-01R. The BSP products were dA-tailed and ligated to Illumina TruSeq adapters, pooled, and sequenced using Illumina MiSeq system. Detailed library preparation procedures are described in Method 4 in S1 Text. Sequencing outputs were minimally trimmed, mapped to genome, and called for methylation rate as aforementioned, except bwa-mem’s “-U” switch was set to its default.

In order to relate the methylation state of the integrated fragments to possible binding or recognition by DNA-binding proteins (e.g., transcription factors), we identified DNase I hypersensitive sites (DHS) by remapping the publicly available DNase-seq dataset of drR medaka blastula embryos (accession number: SRX1032807 [83]) to the medaka genome assembly v2.2.4. Adaptor trimming and alignment was accomplished using BBmap v37.36 [76] with default parameters. Aligned reads were filtered for a minimum MAPQ of 20. MACS v2.1.1.20160309 [84] was subsequently used to called 112987 peaks (DHS) with the following switches: “-g 6.3e+8 --nomodel --shift -50 --extsize 100 -q 0.01”. Vast majority (> 96%) of the assayed fragments were originated completely from either inside or outside-, but not spanning across the boundaries-, of DHS (S3 Table).

### Transplantation of HypoMDs and HyperMDs to specific genomic locus via site-specific recombination

An engineered transgenic line that carries an attP site inside a gene desert on chromosome 18 for PhiC31 integrase-mediated recombination was used for site-specific integration of the full-length, unmethylated HyperMDs (i.e. PCR-amplified, cloned, and without pretreating with M.SssI) and pre-methylated HypoMDs (i.e. PCR-amplified, cloned, and pretreated with M.SssI) with lengths of 300–400 bp.

PhiC31 integrase coding sequence was amplified from pPGK-PhiC31o-bpA (a gift from Philippe Soriano; Addgene plasmid #13795) and attached to SV40 nuclear localization sequence (NLS) using primer pair F4-01 and Phusion polymerase (Thermo Fisher Scientific), then blunt-end-cloned using Zero Blunt PCR Cloning Kit (Thermo Fisher Scientific). Cloning direction and proper coding sequence were checked via Sanger sequencing (by FASMAC Co). PhiC31 integrase mRNA was generated from the constructed template via *in vitro* transcription (Method 5 in S1 Text).

Six HyperMDs (see S1 Dataset) with flanking BSP primer binding sites (for F3-01F and F3-01R) and Dam-sites (downstream of the BSP primer sites) were directly synthesized by Thermo Fisher Scientific and Integrated DNA Technologies (USA) as double-stranded DNA and cloned into the targeting vector pEx_MCS-attBtagRFPt (a gift from Joachim Wittbrodt; Addgene plasmid #48876). Eleven HypoMDs were amplified from drR genomic DNA and extended to include BSP primer binding sites and Dam-sites on both ends using primer sets F4-02 through F4-12, then cloned into the targeting vector pEx_MCS-attBtagRFPt. HyperMD-containing targeting vectors were propagated in *dam*^+^ *E*. *coli* (DH5α) (Thermo Fisher Scientific) and pooled in approximately equimolar amount. HypoMD-containing targeting vectors were similarly processed, except that the pooled library was further artificially methylated with CpG methyltransferase M.SssI and purified as aforementioned (Method 3 in S1 Text). Individual plasmid libraries (final concentration: 10 ng/μL) was injected with PhiC31 integrase mRNA (100 ng/μL) into >200 embryos of PhiC31 transgenic strain [49] at 1-cell stage. Injected embryos were reared at 28°C to blastula stage, screened for normal development (> 85%), homogenized, and extracted for genomic DNA (Method 1 in S1 Text). The extracted DNA was digested with DpnI to degrade unintegrated vectors, re-purified, bisulfite-converted, subjected to PCR via ExTaq polymerase, TA-cloned, Sanger-sequenced, and quantified for methylation rate as aforementioned.

To ensure the injected but unintegrated vectors were efficiently removed, the above injection was also carried out without PhiC31 integrase mRNA. These injected embryos were processed in parallel with those injected with integrase mRNA up to DpnI digestion. The relative abundance of undigested libraries (both unintegrated and integrated) was quantified and normalized to amount of input genomic DNA using real-time PCR as described above (see also S2 Fig: panel B).

### *In vivo* ‘methylome editing’ via homology directed repair

Homology directed repair was triggered by CRISPR-Cas9-induced double-strand breaks. spCas9 mRNA was produced from pMLM3613 (a gift from Keith Joung; Addgene plasmid #42251) via *in vitro* transcription (Method 5 in S1 Text). The HypoMDs, chr17:6415960–6416269 (Locus 1) and chr21:25260707–25262742 (Locus 2), were randomly chosen as targets for editing. sgRNAs targeting these regions were designed using CCTop [85]. The six top-ranked guide sequence designs (sets F5-01 and F5-02, for the two loci, respectively) were synthesized (Thermo Fisher Scientific) and *in vitro* transcribed (Method 5 in S1 Text). To construct the repair template, these genomic regions (with 6 mutations to the targeted spCas9 PAMs, i.e. from ‘NGG’ to ‘NGC’, in order to protect the template from being cleaved by spCas9) along with their up- and down-stream sequences (800 bp on both sides) as homology arms were synthesized (Integrated DNA Technologies), assembled, cloned into pCR-BluntII vector (Thermo Fisher Scientific) using NEBuilder HiFi assembly mix (New England Biolabs), and propagated by *dam*^+^ *E*. *coli*. The repair templates were artificially methylated *in vitro* using CpG methyltransferase M.SssI and purified as described above. For each of the target regions, sgRNA cocktail, spCas9 mRNA, and artificially Dam+CpG-methylated repair template were co-injected into medaka (drR strain) embryos at 1-cell stage at ultra-high concentrations (25 ng/μL each, 600 ng/μL and 10 ng/μL, respectively, i.e. 750 ng/μL of RNA and 10 ng/μL DNA in total) to maximize editing rate. Injected embryos were reared at 28°C for approx. 8 hours to blastula stage, screened for normal development (> 75%) and extracted for genomic DNA, which was DpnI-treated to degrade the repair template, re-purified, and bisulfite-converted as aforementioned. The BSP primer pairs (F5-03 for Locus 1; F5-04 for Locus 2) were designed using MethPrimer 2.0 and screened for the presence of native Dam-site(s) (5’-GATC-3’) within the target region. The amplification products were gel-purified and directly Sanger-sequenced from both ends. The methylation rate of each CpG was estimated from the sequencing chromatograms as: C ÷ (C + T) × 100%, where C and T are the called peak height in the ‘cytosine’ (i.e. methylated cytosines, after bisulfite PCR) and ‘thymine’ (i.e. unmethylated cytosines, which were converted to uracil by bisulfite treatment, then to thymine by PCR) channels, respectively. The signal intensities were extracted in R 3.3.3 [86] using the sangerseqR package (version 1.12.0) [87]. To estimate the editing rate, regions containing the sgRNA target sites was PCR-amplified from unconverted DNA using primer sets F5-05 through F5-09. Editing rate was gauged by the relative frequency of mutated sgRNA PAMs (5’-NGC-3’; on the edited alleles) versus the native PAMs (5’-NGG-3’; i.e. unedited alleles) from the Sanger sequencing trace using the same approach as described above. Editing efficiency was estimated to be 92.04% and 85.10% for Locus 1 and 2, respectively.

To collect edited embryos at later developmental stages (3 and 7 day-post-fertilization; dpf), the above cocktail was diluted 10-fold (in Milli-Q water; Merck Millipore) immediately prior to injection to reduce the toxicity (manifested after gastrulation) of ultra-high nuclei acid concentration at the expense of efficient editing. DNA extraction and subsequent processing were carried as above. Estimated editing efficiency for Locus 1 = 9.56% (at 3 dpf) and 7.81% (at 7 dpf); Locus 2 = 27.16% (at 3 dpf) and 18.69% (at 7 dpf). In order to enable comparison across sampling time-points with variable editing rates, the estimated methylation rates were normalized to the editing efficiency (i.e. “normalized methylation rate” = “methylation rate” ÷ “editing rate”). Raw values prior to normalization are available in S1 Dataset.

## Supporting information

S1 Fig Violin plots showing the distribution of (A) length, (B) GC content, and (C) CpG density of HypoMDs and HyperMDs.
(TIF)

S2 Fig Efficient removal of injected but unintegrated libraries via PEG precipitation and DpnI digestion. (A) MspI-captured fragments with or without pre-methylation via CpG methyltransferase M.SssI. Since it is technically infeasible to prevent the spontaneous integration of linear DNA, an integration-free surrogate control (“2× spike-in”) was generated by spiking-in the injection mixtures directly into fresh lysate of uninjected blastula embryos. Approximately twice the amount of the injection mix consumed by genuinely injected embryos (“Injected”), i.e. ca. 34 pL per embryo, was spiked-in to provide conservative estimation of the removal efficiency of unintegrated fragments. (B) Plasmid libraries containing unmethylated HyperMDs or M.SssI-methylated HypoMDs. Since spontaneous integration of circular DNA (i.e. plasmids) into the genome is generally very rare in the absence of integrase, integration-free surrogate control (“- integrase”) was generated by injecting PhiC31 medaka embryos without PhiC31 integrase mRNA (in contrast to 100 ng/μL of the mRNA for the integration experiment, i.e. “+ integrase”). Error bars represent 95% confidence intervals.
(TIF)

S3 Fig Violin plots showing the distribution of (A) length, (B) GC content, and (C) CpG density of the unmethylated or pre-methylated fragments that were successfully integrated into genome and subsequently assayed.
(TIF)

S4 Fig Sampling origins (from HypoMDs, HyperMDs, or elsewhere in the genome) of the assayed CpGs on the integrated genomic fragments. Note that CpGs from HypoMDs and HyperMDs were nearly equally represented (upper panels vs lower panels).
(TIF)

S5 Fig **Correlation between the methylation rates of the same CpGs at their endogenous versus at reintegrated/ectopic positions, (A) without- or (B) with artificial methylation prior to injection and genome integration.** The methylation rates were bimodal and strongly skewed towards either 0% or 100%. To circumvent over-plotting, individual CpGs, *N* = (A) 10251 and (B) 18537, were consolidated into hexes (bin width = 1%), with the shade of the hex representing the number of CpGs included (in logarithmic scale). Bars on the top and right-hand side of each of the scatterplots are the histograms that show the density of CpGs along the corresponding axes (bin width = 1%). Correlation coefficients: Spearman’s ρ = (A) 0.08, (B) 0.02; Kendall’s τ = (A) 0.07, (B) 0.01.
(TIF)

S6 Fig Differential sensitivity of untreated and M.SssI-treated DNA samples to MspI and HpaII restriction enzymes. (A) *E*. *coli* genomic DNA. (B) Plasmid library that was used to generate results shown in Fig 4D. Approximate CpG and MspI/HpaII restriction site densities (counts per kilobase pair): (A) 76 and 5, (B) 62 and 4, which are much higher than those in medaka genome, i.e. 23 and 1. Note that there is no observable cleavage by HpaII after pretreatment with the methyltransferase, suggesting complete methylation was achieved using our reaction regimen. “L”: Thermo Fisher Scientific 1Kb Plus DNA ladder. “Mock”: control reaction without restriction enzyme.
(TIF)

S7 Fig Correlation of CpG methylation state at ectopic versus native positions with respect to the endogenous local chromatin accessibility. The biplots are alternative representation of S5 Fig. CpGs from the inside and outside of DNaseI hypersensitive sites (DHS) were graphed separately (i.e. upper versus lower panels). To circumvent over-plotting, fragments sharing similar methylation states were consolidated into hexes (bin width = 1%), with the shade of the hexes representing the number of fragments included (in logarithmic scale). Numerical figures denoted on the top of each of the biplots are the correlation coefficients. ρ = Spearman’s rho; τ = Kendall’s tau.
(TIF)

S8 Fig Precision-recall curve of kmer-SVMs for classification of CpGs and their flanking sequences that underwent demethylation. Demethylated (*N* = 23655) and hypermethylated (*N* = 30760) sequences (including 10 bp from both up- and down-stream of the CpG) were assigned to positive and negative classes, respectively. Solid, colored lines are individual precision-recall curves derived from 10-fold cross-validation. The colors represent the cut-off values for binary classification/prediction of the testing pool in each rounds of cross-validation. Area-under-curve (AUC): minimum = 0.46, maximum = 0.47. Random classifier is represented by the horizontal dashes at the center and has an AUC of 0.43.
(TIF)

S9 Fig **Correlation between the overall methylation rate and (A) length or (B) CpG density of the integrated fragments.** Integrated fragments derived from the (left) unmethylated or (right) M.SssI-treated libraries were further segregated according to their endogenous methylation state (top vs bottom). Fragments are defined as endogenously (top) hypomethylated or (bottom) hypermethylated if they have a mean CpG-methylation rate of < 40% or > 60%, respectively. To circumvent over-plotting, fragments with similar methylation rate and (A) length or (B) CpG density were consolidated into hexes (number of bins = 100, both horizontally and vertically), with the shade of the hexes representing the number of fragments included (in logarithmic scale). “ρ” and “τ” indicate the Spearman’s rho and Kendall’s tau correlation coefficients of the corresponding scatterplots.
(TIF)

S10 Fig Methylation state of the *in situ* edited HypoMDs at multiple embryonic stages. Edited embryos were sampled at early (blastula, 0 day-post-fertilization; dpf), mid (3 dpf), and late (7 dpf; hatching) embryonic stages. To enable comparison across sampling time-points with variable editing efficiency, the estimated methylation rates were normalized against the estimated editing rate (see Materials and Methods).
(TIF)

S1 Table The top weighted 6-mers from the kmer-SVM trained for classification of HypoMDs and HyperMDs. The 6-mers are sorted according to their absolute weight, i.e. importance and enrichment, in descending order. CpG dyads are colored red.
(DOCX)

S2 Table List of DNA oligos used. (XLSX)

S3 Table Endogenous origins of the integrated fragments from (left) unmethylated and (right) artificially methylated library. Note that vast majority (> 96%) of the assayed fragments were derived completely from either inside or outside-, but not spanning across the boundaries of-, DNase I hypersensitive sites (DHS).
(DOCX)

S1 Text Supplementary methods. (DOCX)

S1 Dataset Raw data. (ZIP)
