# Supplementary figures and images for: Unlinking the methylome pattern from nucleotide sequence, revealed by large-scale in vivo genome engineering and methylome editing in medaka fish
Source: PLoS Genet. 2017 Dec 21;13(12):e1007123. doi: 10.1371/journal.pgen.1007123 (PMC5755920; doi:10.1371/journal.pgen.1007123)

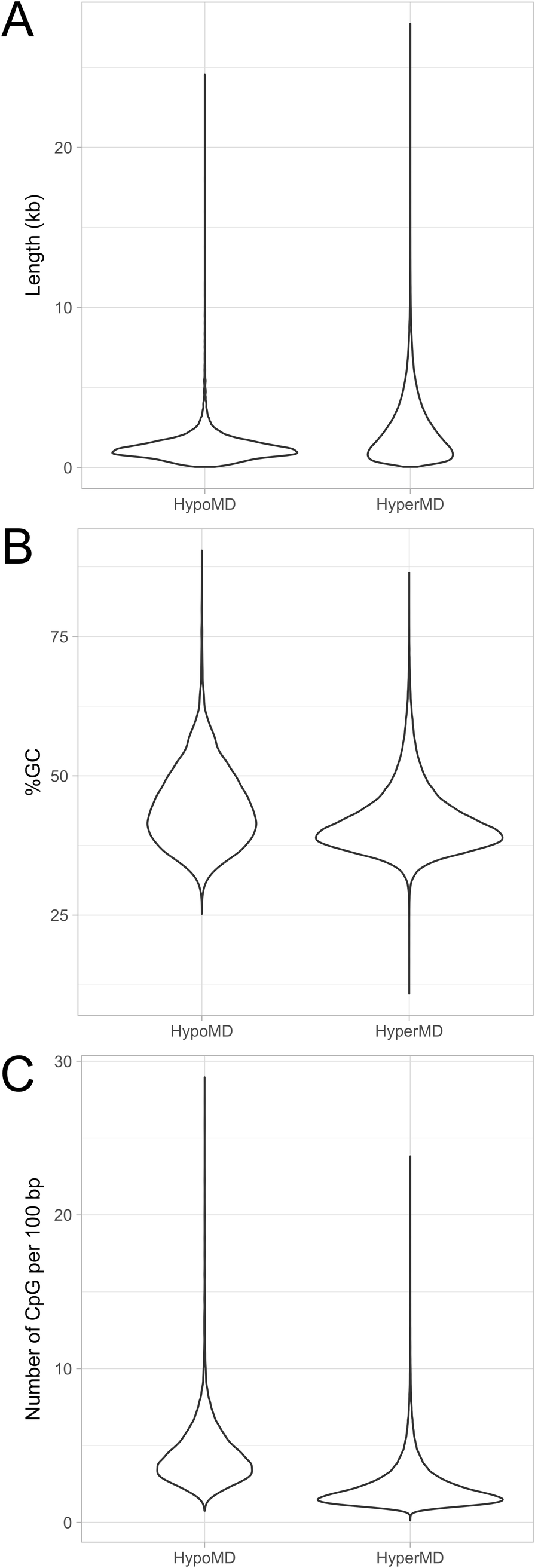

Supplement: S1 Fig — Violin plots showing the distribution of (A) length, (B) GC content, and (C) CpG density of HypoMDs and HyperMDs. (TIF) [file pgen.1007123.s001.tif]

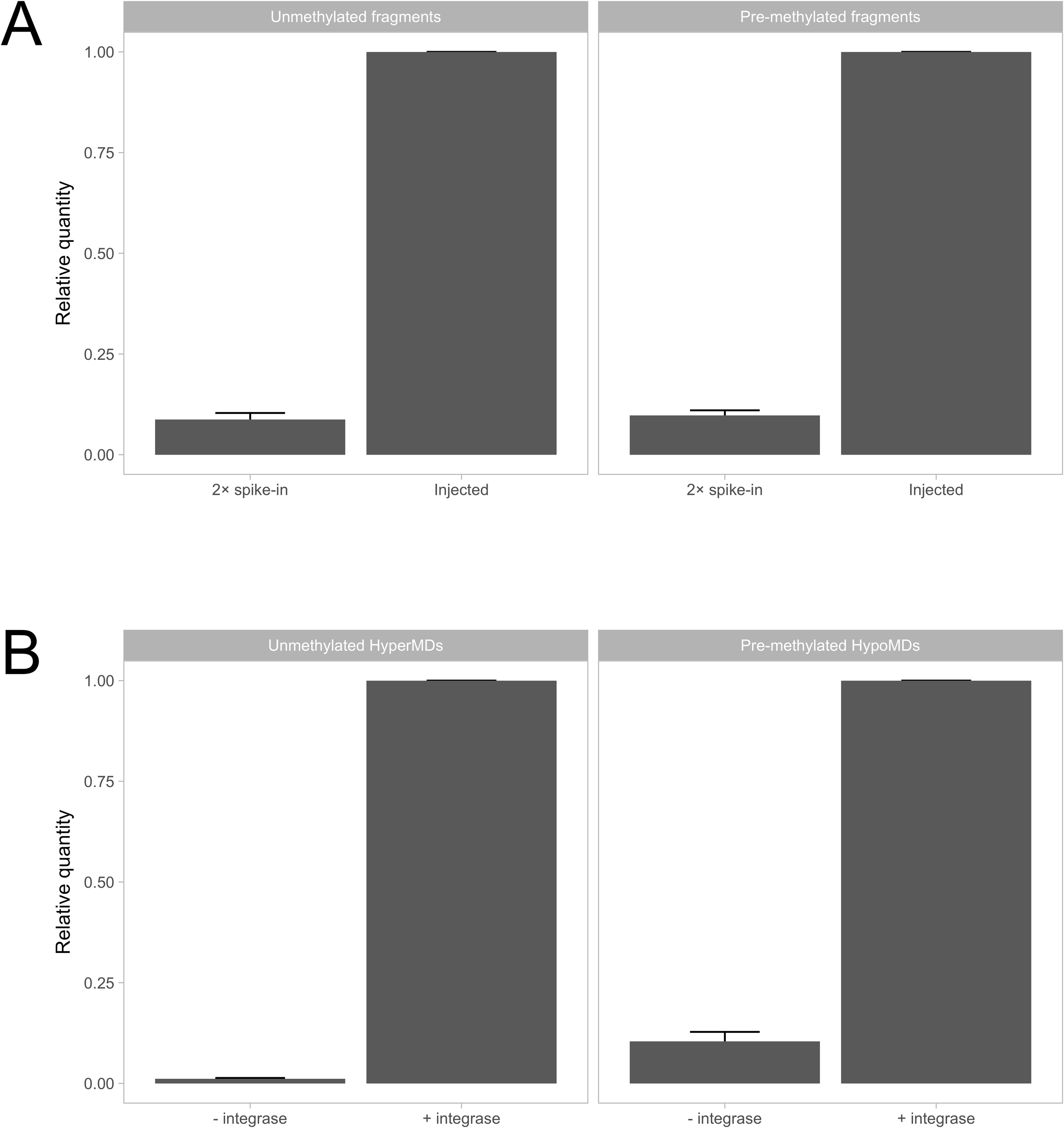

Supplement: S2 Fig — (A) MspI-captured fragments with or without pre-methylation via CpG methyltransferase M.SssI. Since it is technically infeasible to prevent the spontaneous integration of linear DNA, an integration-free surrogate control (“2× spike-in”) was generated by spiking-in the injection mixtures directly into fresh lysate of uninjected blastula embryos. Approximately twice the amount of the injection mix consumed by genuinely injected embryos (“Injected”), i.e. ca. 34 pL per embryo, was spiked-in to provide conservative estimation of the removal efficiency of unintegrated fragments. (B) Plasmid libraries containing unmethylated HyperMDs or M.SssI-methylated HypoMDs. Since spontaneous integration of circular DNA (i.e. plasmids) into the genome is generally very rare in the absence of integrase, integration-free surrogate control (“- integrase”) was generated by injecting PhiC31 medaka embryos without PhiC31 integrase mRNA (in contrast to 100 ng/μL of the mRNA for the integration experiment, i.e. “+ integrase”). Error bars represent 95% confidence intervals. (TIF) [file pgen.1007123.s002.tif]

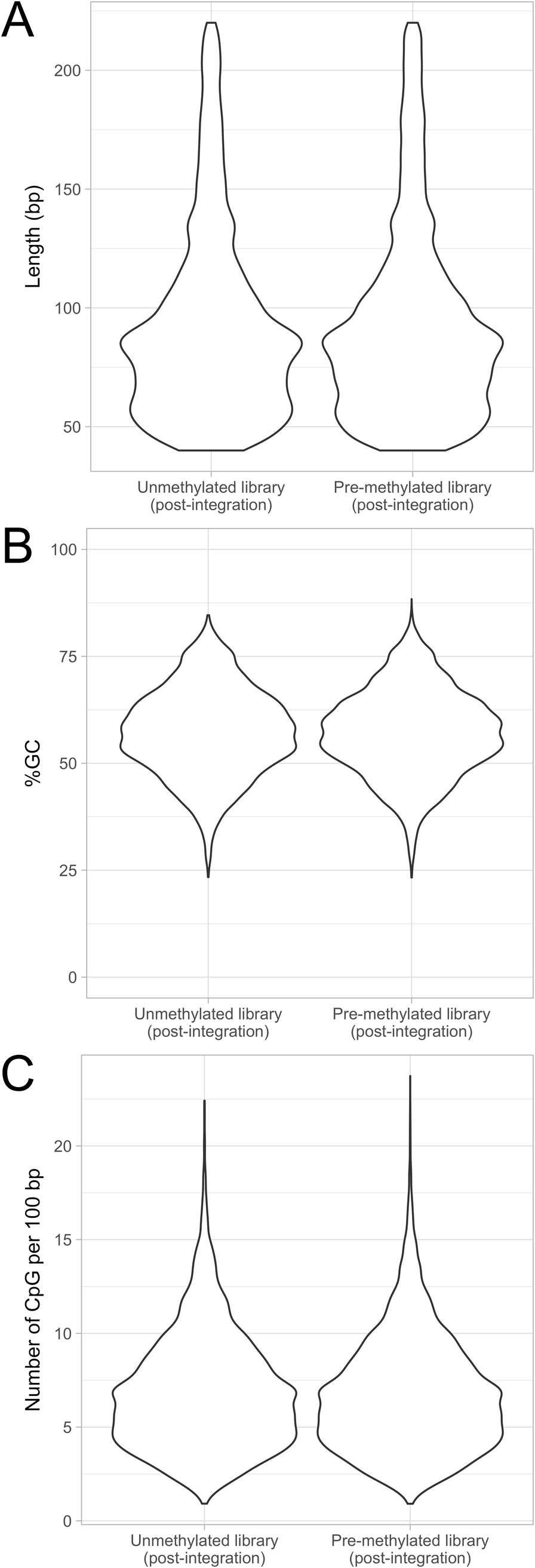

Supplement: S3 Fig — Violin plots showing the distribution of (A) length, (B) GC content, and (C) CpG density of the unmethylated or pre-methylated fragments that were successfully integrated into genome and subsequently assayed. (TIF) [file pgen.1007123.s003.tif]

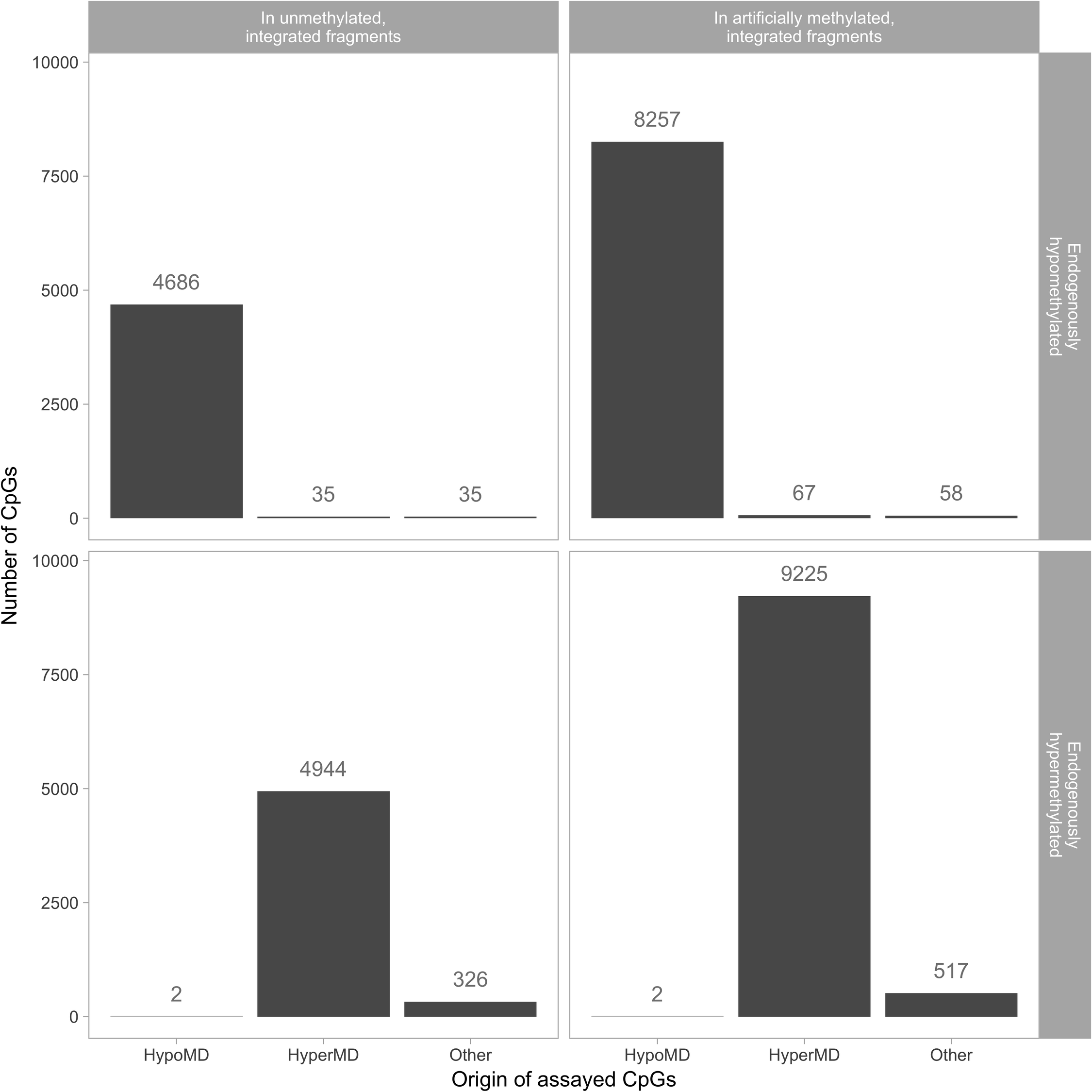

Supplement: S4 Fig — Note that CpGs from HypoMDs and HyperMDs were nearly equally represented (upper panels vs lower panels). (TIF) [file pgen.1007123.s004.tif]

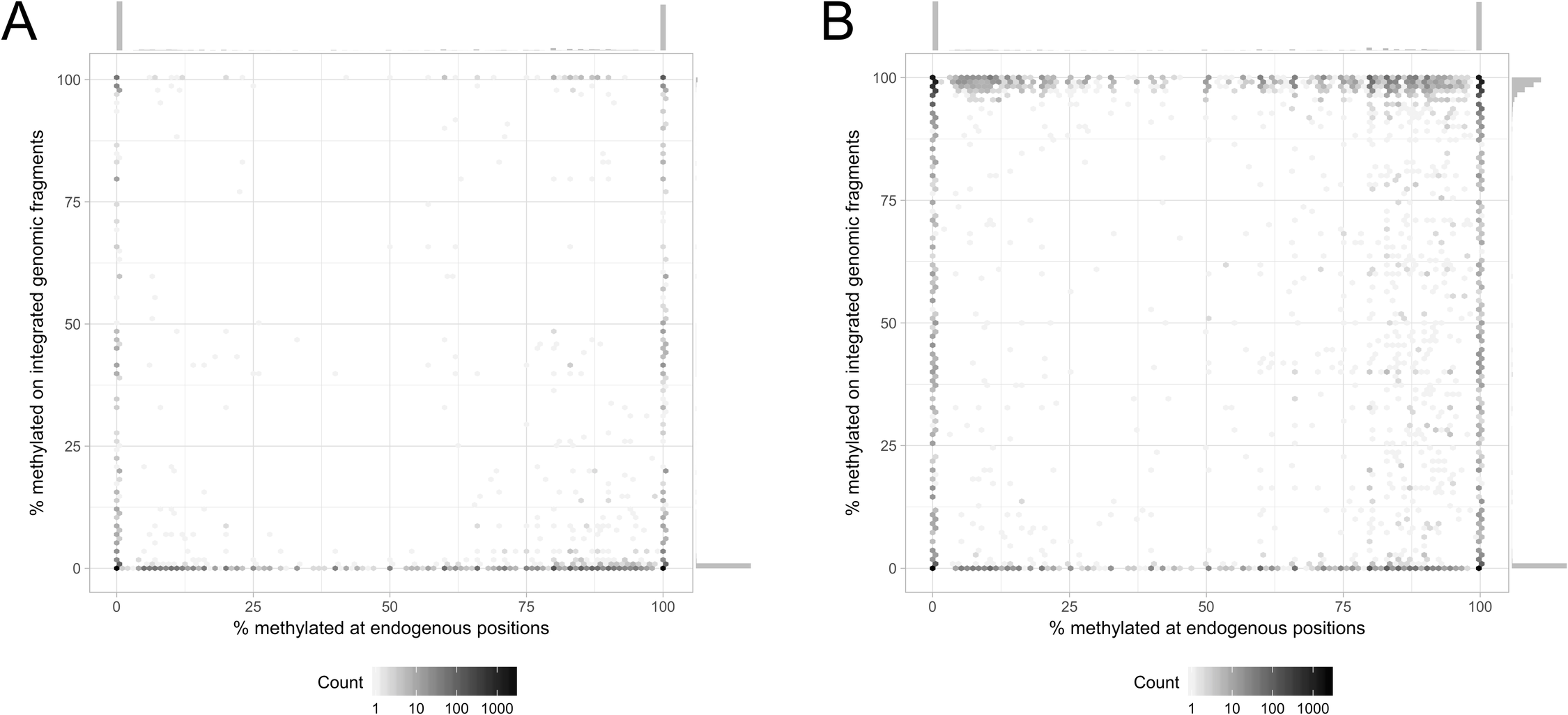

Supplement: S5 Fig — Correlation between the methylation rates of the same CpGs at their endogenous versus at reintegrated/ectopic positions, (A) without- or (B) with artificial methylation prior to injection and genome integration. The methylation rates were bimodal and strongly skewed towards either 0% or 100%. To circumvent over-plotting, individual CpGs, N = (A) 10251 and (B) 18537, were consolidated into hexes (bin width = 1%), with the shade of the hex representing the number of CpGs included (in logarithmic scale). Bars on the top and right-hand side of each of the scatterplots are the histograms that show the density of CpGs along the corresponding axes (bin width = 1%). Correlation coefficients: Spearman’s ρ = (A) 0.08, (B) 0.02; Kendall’s τ = (A) 0.07, (B) 0.01. (TIF) [file pgen.1007123.s005.tif]

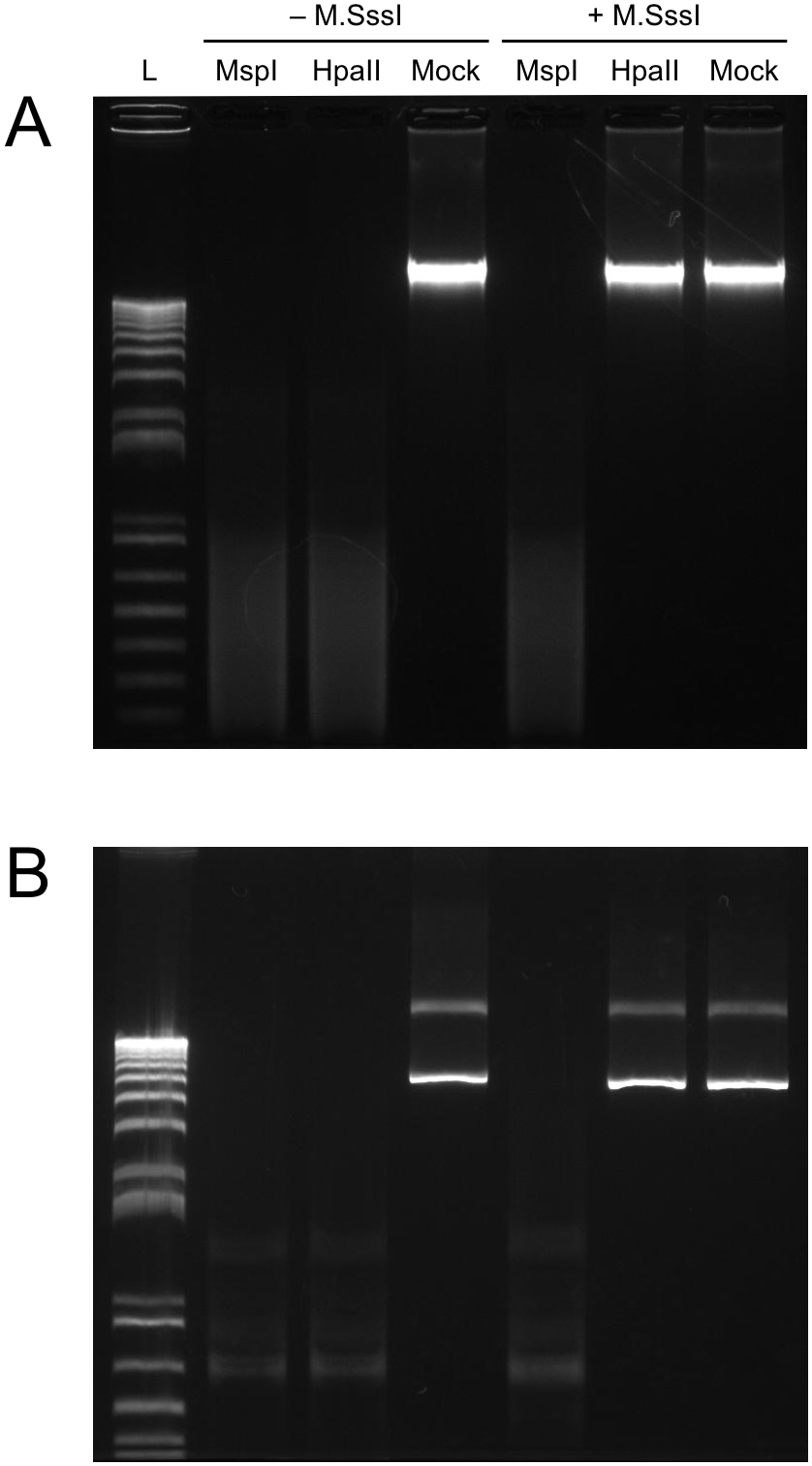

Supplement: S6 Fig — (A) E. coli genomic DNA. (B) Plasmid library that was used to generate results shown in Fig 4D. Approximate CpG and MspI/HpaII restriction site densities (counts per kilobase pair): (A) 76 and 5, (B) 62 and 4, which are much higher than those in medaka genome, i.e. 23 and 1. Note that there is no observable cleavage by HpaII after pretreatment with the methyltransferase, suggesting complete methylation was achieved using our reaction regimen. “L”: Thermo Fisher Scientific 1Kb Plus DNA ladder. “Mock”: control reaction without restriction enzyme. (TIF) [file pgen.1007123.s006.tif]

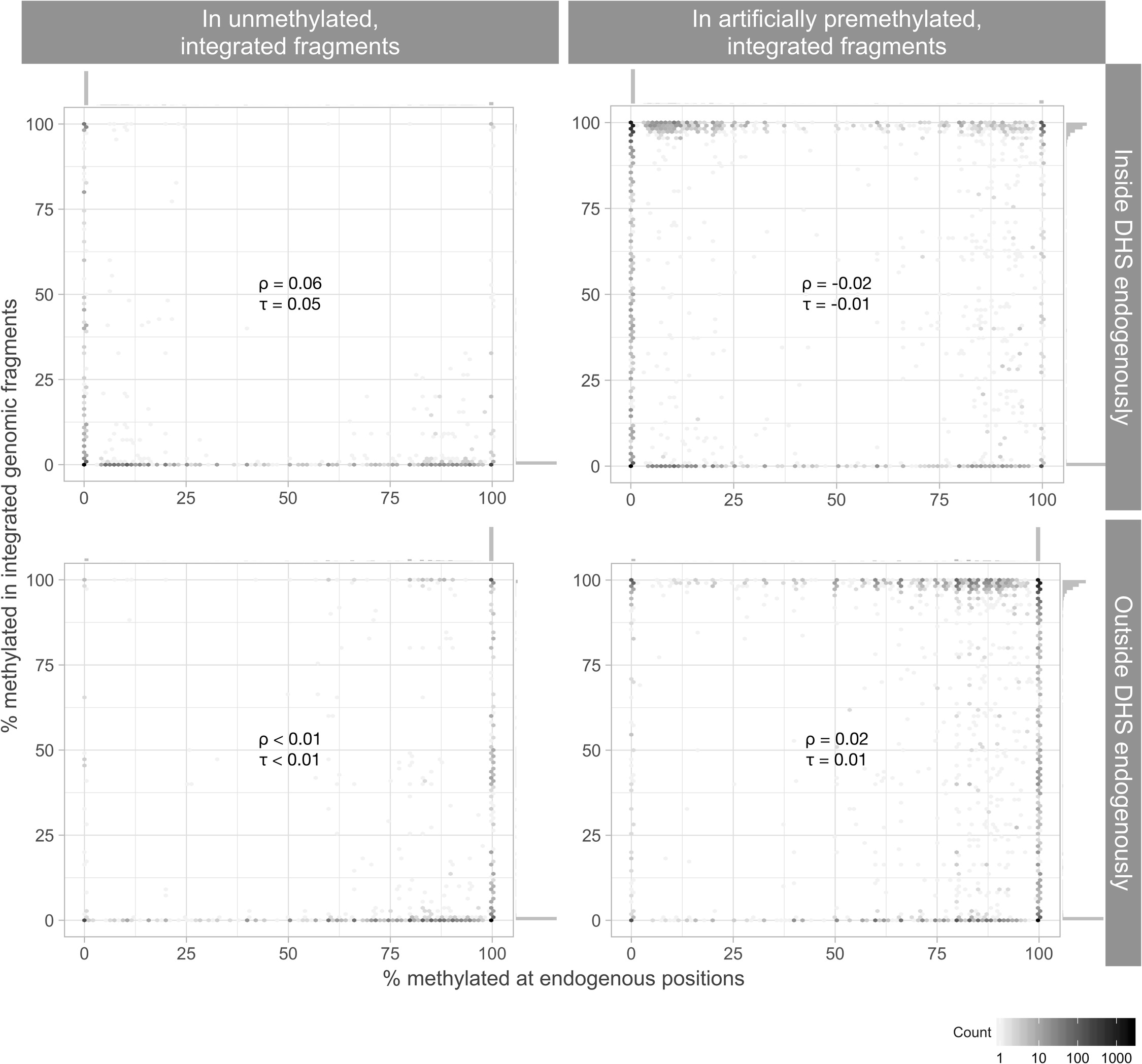

Supplement: S7 Fig — The biplots are alternative representation of S5 Fig. CpGs from the inside and outside of DNaseI hypersensitive sites (DHS) were graphed separately (i.e. upper versus lower panels). To circumvent over-plotting, fragments sharing similar methylation states were consolidated into hexes (bin width = 1%), with the shade of the hexes representing the number of fragments included (in logarithmic scale). Numerical figures denoted on the top of each of the biplots are the correlation coefficients. ρ = Spearman’s rho; τ = Kendall’s tau. (TIF) [file pgen.1007123.s007.tif]

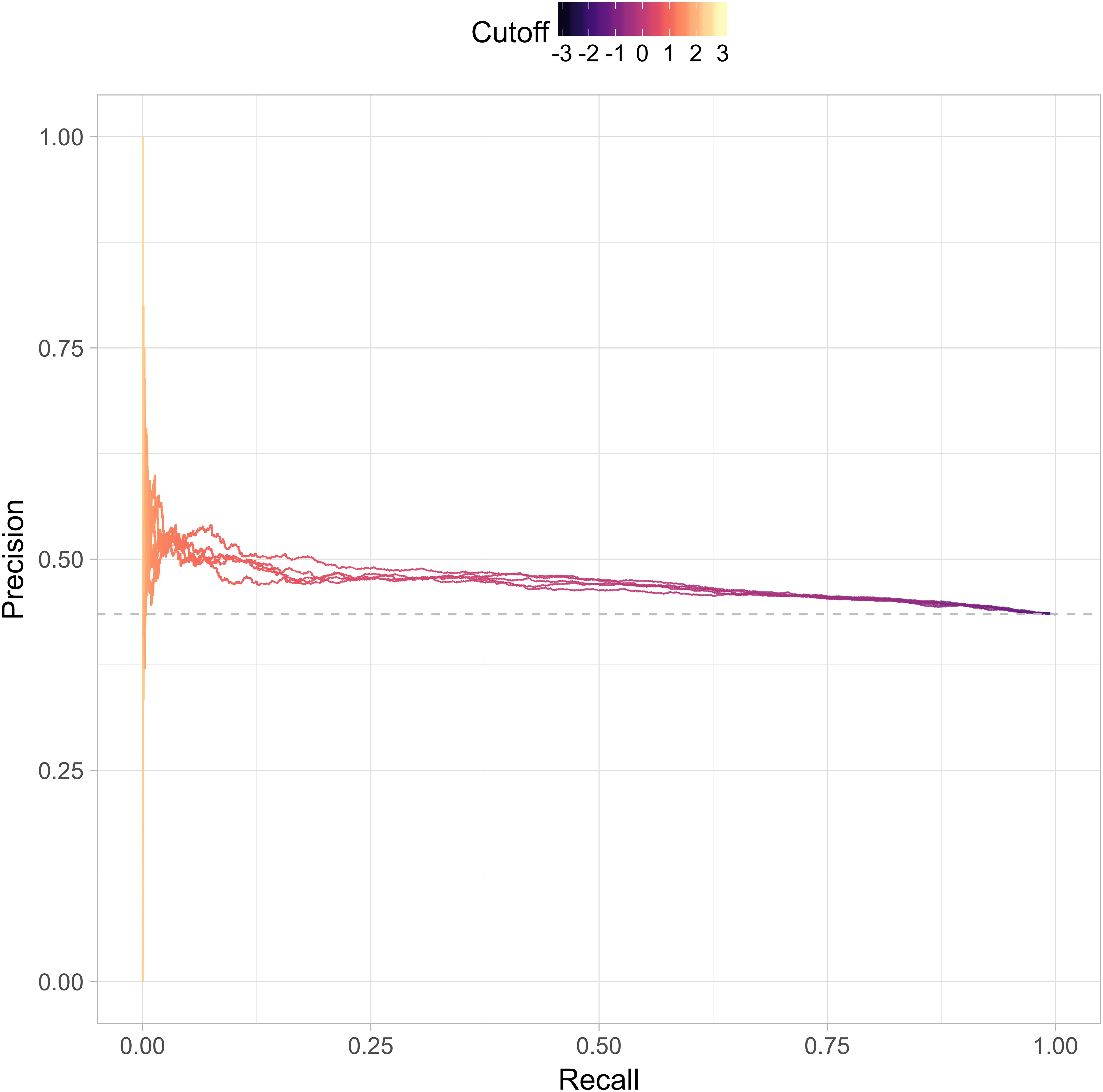

Supplement: S8 Fig — Demethylated (N = 23655) and hypermethylated (N = 30760) sequences (including 10 bp from both up- and down-stream of the CpG) were assigned to positive and negative classes, respectively. Solid, colored lines are individual precision-recall curves derived from 10-fold cross-validation. The colors represent the cut-off values for binary classification/prediction of the testing pool in each rounds of cross-validation. Area-under-curve (AUC): minimum = 0.46, maximum = 0.47. Random classifier is represented by the horizontal dashes at the center and has an AUC of 0.43. (TIF) [file pgen.1007123.s008.tif]

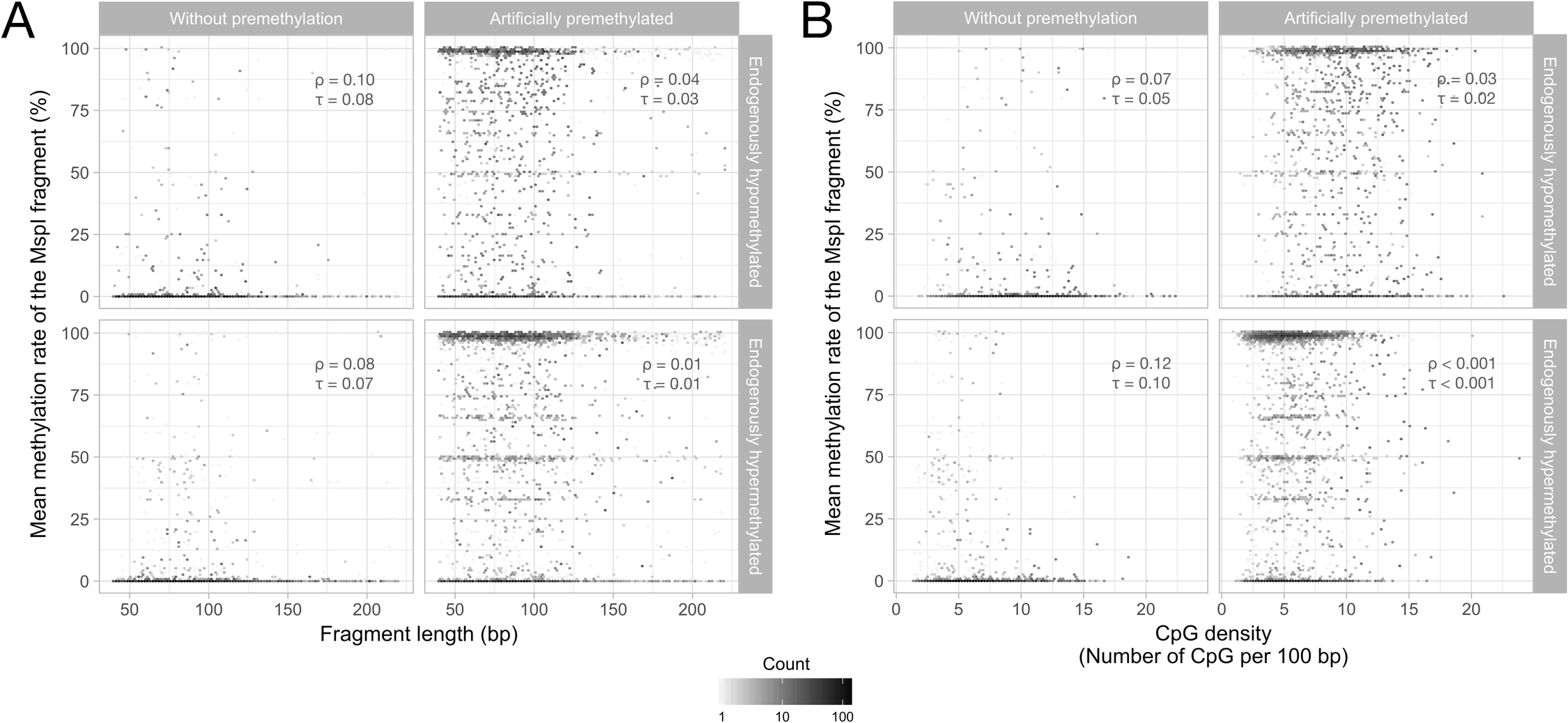

Supplement: S9 Fig — Correlation between the overall methylation rate and (A) length or (B) CpG density of the integrated fragments. Integrated fragments derived from the (left) unmethylated or (right) M.SssI-treated libraries were further segregated according to their endogenous methylation state (top vs bottom). Fragments are defined as endogenously (top) hypomethylated or (bottom) hypermethylated if they have a mean CpG-methylation rate of < 40% or > 60%, respectively. To circumvent over-plotting, fragments with similar methylation rate and (A) length or (B) CpG density were consolidated into hexes (number of bins = 100, both horizontally and vertically), with the shade of the hexes representing the number of fragments included (in logarithmic scale). “ρ” and “τ” indicate the Spearman’s rho and Kendall’s tau correlation coefficients of the corresponding scatterplots. (TIF) [file pgen.1007123.s009.tif]

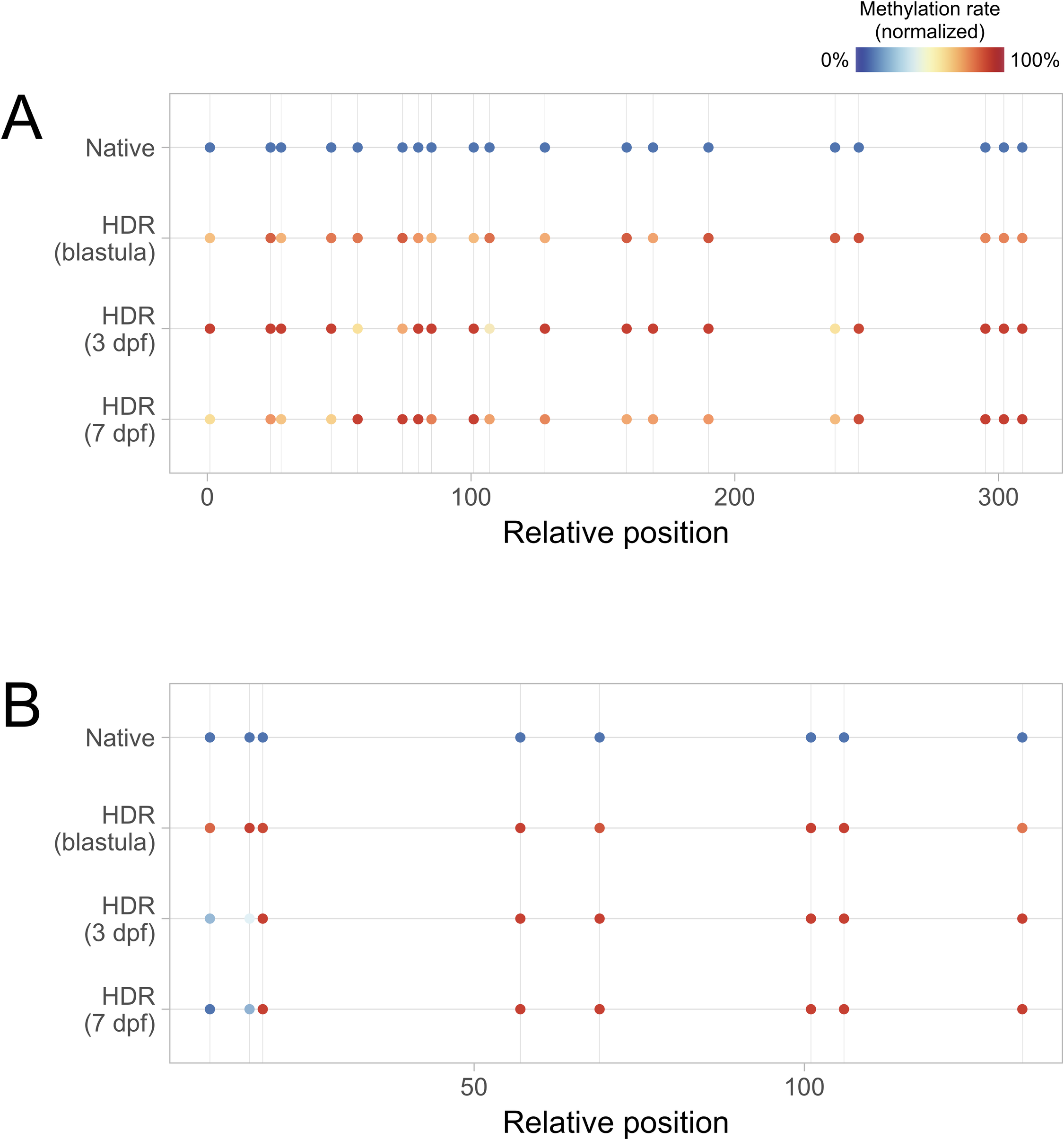

Supplement: S10 Fig — Edited embryos were sampled at early (blastula, 0 day-post-fertilization; dpf), mid (3 dpf), and late (7 dpf; hatching) embryonic stages. To enable comparison across sampling time-points with variable editing efficiency, the estimated methylation rates were normalized against the estimated editing rate (see Materials and Methods). (TIF) [file pgen.1007123.s010.tif]

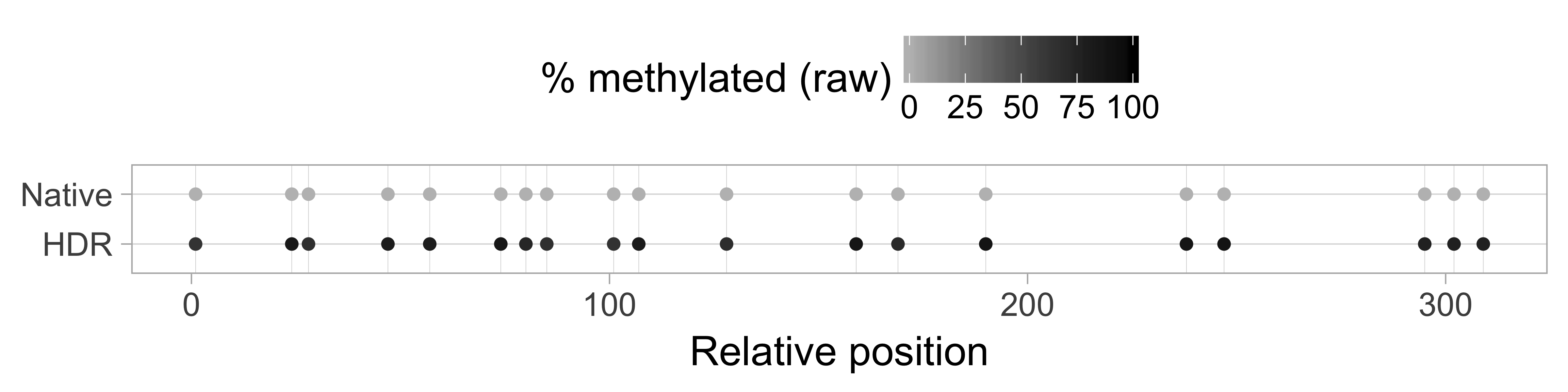

Supplement: S1 Dataset — (ZIP) [file pgen.1007123.s015.zip › S1_Dataset/S9_Fig_A_lollipop_0dpf_unnormalized.png]

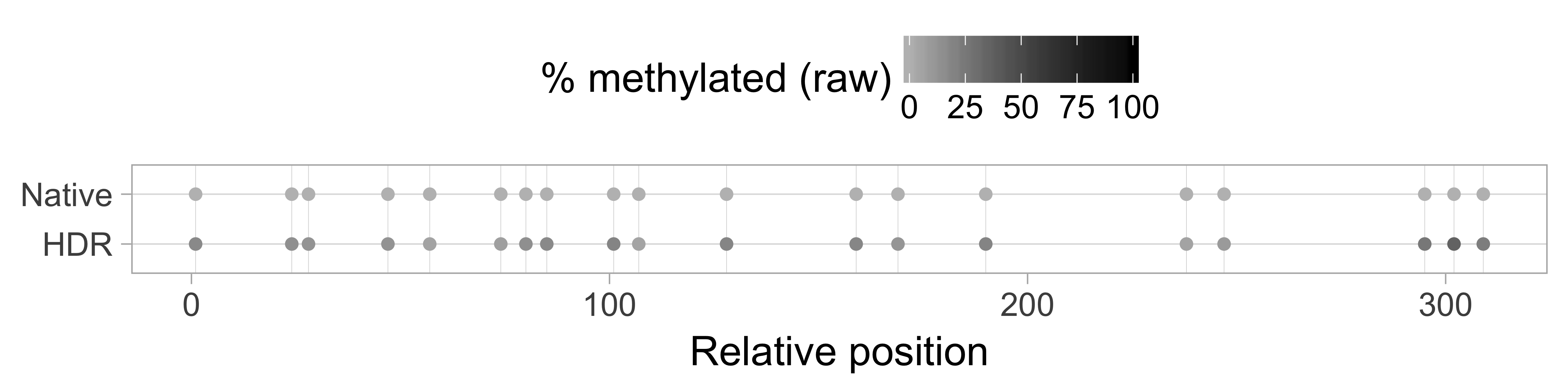

Supplement: S1 Dataset — (ZIP) [file pgen.1007123.s015.zip › S1_Dataset/S9_Fig_A_lollipop_3dpf_unnormalized.png]

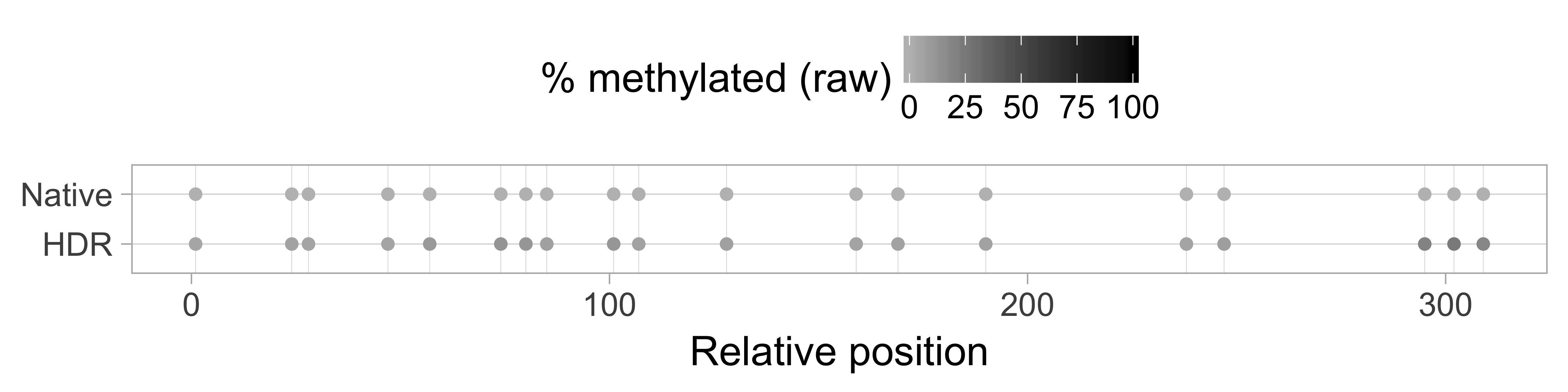

Supplement: S1 Dataset — (ZIP) [file pgen.1007123.s015.zip › S1_Dataset/S9_Fig_A_lollipop_7dpf_unnormalized.png]

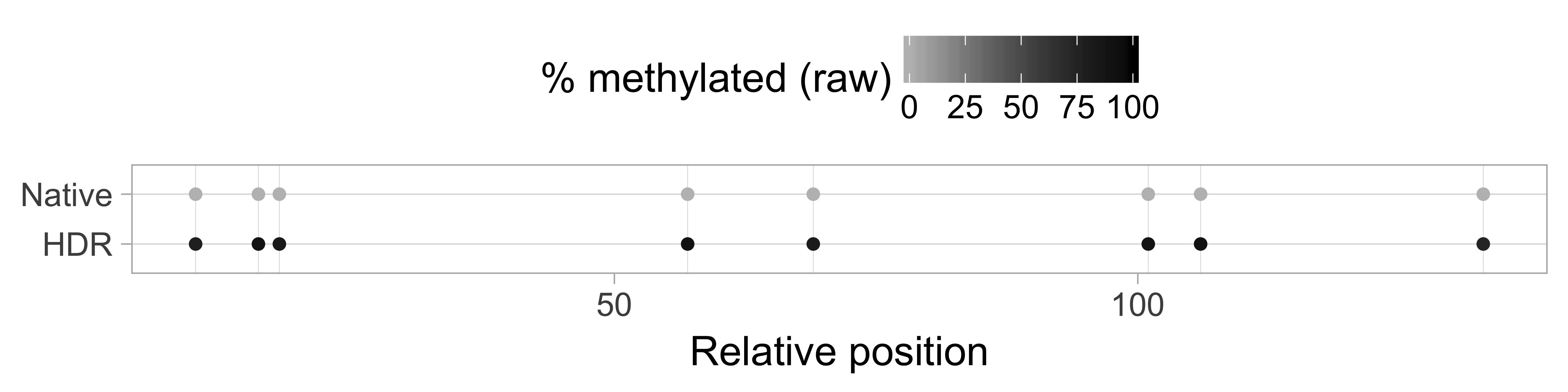

Supplement: S1 Dataset — (ZIP) [file pgen.1007123.s015.zip › S1_Dataset/S9_Fig_B_lollipop_0dpf_unnormalized.png]

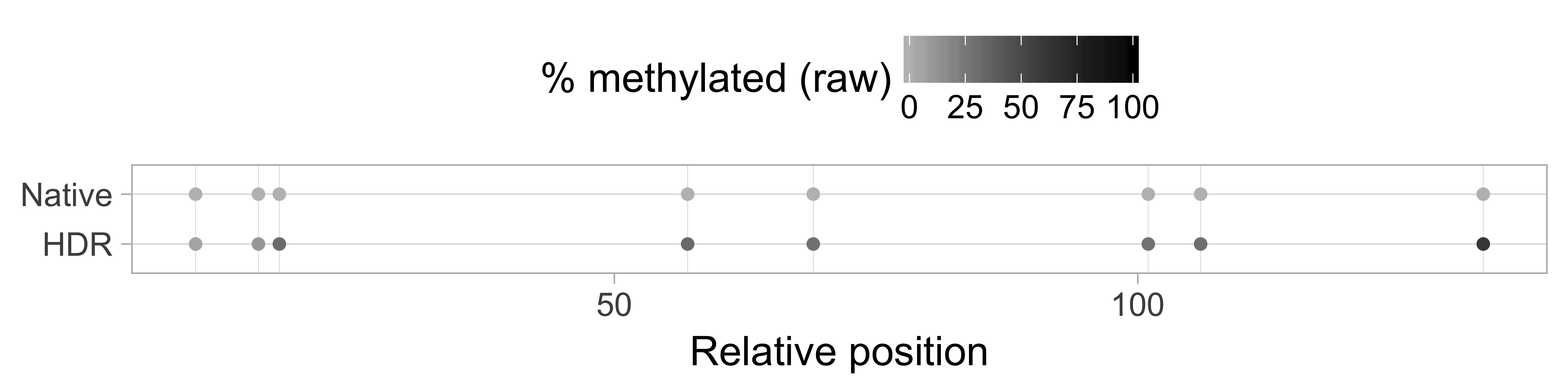

Supplement: S1 Dataset — (ZIP) [file pgen.1007123.s015.zip › S1_Dataset/S9_Fig_B_lollipop_3dpf_unnormalized.png]

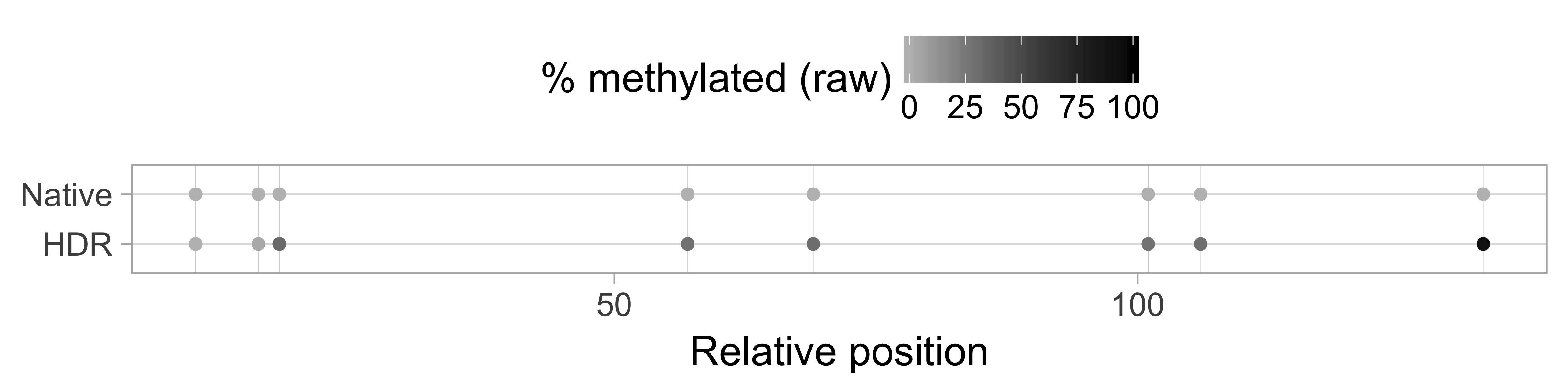

Supplement: S1 Dataset — (ZIP) [file pgen.1007123.s015.zip › S1_Dataset/S9_Fig_B_lollipop_7dpf_unnormalized.png]
