# Supplementary material for: Unlinking the methylome pattern from nucleotide sequence, revealed by large-scale in vivo genome engineering and methylome editing in medaka fish
Source: PLoS Genet. 2017 Dec 21;13(12):e1007123. doi: 10.1371/journal.pgen.1007123 (PMC5755920; doi:10.1371/journal.pgen.1007123)
Supplement: S1 Table — The 6-mers are sorted according to their absolute weight, i.e. importance and enrichment, in descending order. CpG dyads are colored red. (DOCX) [file pgen.1007123.s011.docx]

**S1 Table The top weighted 6-mers from the kmer-SVM trained for classification of HypoMDs and HyperMDs.** The 6-mers are sorted according to their absolute weight, i.e. importance and enrichment, in descending order. CpG dyads are colored red.

| Without CpG-masking | | CpG-masked | |
| --- | --- | --- | --- |
| Weighed towards HypoMD | Weighed towards HyperMD | Weighed towards HypoMD | Weighed towards HyperMD |
| GCGCGC | CAACCG | GCTAGC | AAATTT |
| AAAACG | ATACGC | AGCTAG | AAATTG |
| ACGCGC | GGCCCA | TAGCTA | AAAAAT |
| CGCGCG | ATCGCA | GCTAAC | AAATTC |
| ATGCGC | CACTAC | CAAAAA | AAGCTT |
| CGCGAG | AAACGC | ACTTAC | ATATTG |
| CGTTTA | CTCGCC | AGCTAA | CCCTAG |
| CGCGGA | AAACGT | GAAAAA | GCTAGA |
| GCGCGA | AAACGG | ACTCAC | AAAAAC |
| CGTTTC | ACGCCA | CTTACC | GCCCTA |
| TGCGCA | AGCGTA | AAGAAG | AAAAAG |
| CAAACG | CCCCCA | TTTAAA | AAGGCC |
| GCTAGC | CCCTAG | CTAGCA | AAGCAC |
| GCGCAC | AGAGCG | AATTTA | CATGTA |
| CGCGCA | AACCGT | AGGTAA | GAGCTA |
| CGGAAG | ACCGTA | TTAAAA | GACATA |
| ACGCGG | AAGGCC | ATCATG | ACTATG |
| TACGCA | GCTCTA | TAAAAA | GCTCTA |
| GCGTAA | CGCCTA | CTAAAA | CACTAC |
| AGCTAG | GGGTCA | AGGGGG | AGGTCA |
