# Supplementary material for: Unlinking the methylome pattern from nucleotide sequence, revealed by large-scale in vivo genome engineering and methylome editing in medaka fish
Source: PLoS Genet. 2017 Dec 21;13(12):e1007123. doi: 10.1371/journal.pgen.1007123 (PMC5755920; doi:10.1371/journal.pgen.1007123)
Supplement: S3 Table — Note that vast majority (> 96%) of the assayed fragments were derived completely from either inside or outside-, but not spanning across the boundaries of-, DNase I hypersensitive sites (DHS). (DOCX) [file pgen.1007123.s013.docx]

**S3 Table Endogenous origins of the integrated fragments from (left) unmethylated and (right) artificially methylated library.** Note that vast majority (> 96%) of the assayed fragments were completely derived from either inside or outside DNase I hypersensitive sites (DHS).

| Origin | Unmethylated | Pre-methylated |
| --- | --- | --- |
| Outside DHS | 7239  (78.7%) | 12791  (79.3%) |
| Inside DHS | 1622  (17.6%) | 2775  (17.2%) |
| Across edges of DHS | 332  (3.6%) | 568  (3.5%) |
| *Total* | 9193 | 16134 |
