# Supplementary material for: Unlinking the methylome pattern from nucleotide sequence, revealed by large-scale in vivo genome engineering and methylome editing in medaka fish
Source: PLoS Genet. 2017 Dec 21;13(12):e1007123. doi: 10.1371/journal.pgen.1007123 (PMC5755920; doi:10.1371/journal.pgen.1007123)
Supplement: S1 Text — (DOCX) [file pgen.1007123.s014.docx]

**Method 1 Genomic DNA extraction from medaka embryos**

Up to 20 embryos were dispensed into individual 1.5 mL microfuge tubes and were homogenized in 200 µL of 2X CTAB extraction buffer containing 2% CTAB (cetrimonium bromide) (Wako Pure Chemical Industries, Japan), 100 mM Tris-HCl (pH 8), 20 mM EDTA, and 1.4 M NaCl, supplemented with 100 µg/mL of proteinase K, and incubated at 65^o^C overnight. The product was extracted with an equal volume of chloroform. Up to four aqueous layers were pooled to minimize pipetting. CTAB was replenished by the addition of 0.1X volume of 10% CTAB solution (pre-heated to 65^o^C to re-dissolve any CTAB precipitate). The mixture was mixed gently by inversion and re-extracted with an equal volume of chloroform. Nucleic acids were precipitated by mixing with 0.5X vol. of SPRI buffer (20% PEG6000, 2.5 M NaCl, 10 mM Tris-HCl, 1 mM EDTA, 0.05% Tween-20, pH 8) and pelleted at 17900 x g, 25^o^C for 30 mins, desalt by washing with 70% ethanol after complete removal of the supernatant, re-spun, air-dried, and re-dissolved in 10 µL freshly dispensed Milli-Q water. Dissolution was carried out at 65^o^C for ca. 15 mins in an incubator, then cooled on ice. For the removal of injected but unintegrated DNA, the purified nucleic acids were subjected to DpnI digestion and further purified using routine phenol-chloroform and isopropanol precipitation. Prior to bisulfite conversion and/or PCR, RNA degradation was carried out by the addition of RNase A (Wako Pure Chemical Industries) to a final concentration of 1 µg/µL and incubation at 37^o^C for 60 mins. To check the DNA integrity and quantity, 0.5 µL of the extracted genomic DNA was electrophoresized in 1% agarose gel, 0.5X TAE. The purified DNA was immediately subjected to downstream enzymatic treatment and/or bisuflite conversion without being stored frozen. Under these conditions, all of the purified sample DNA achieved bisulfite conversion rate of about 99%.

**Method 2 High-throughput capturing of CpG-rich genomic fragments**

Adult medaka (drR strain; ca. 14-month-old) was anesthetized in ice-cold water and decapitated. Muscles were scraped from the body trunk using scalpel. The muscles were ground to slurry in ca. 500 µL of lysis buffer (100 mM Tris-HCl, 50 mM EDTA, and 1% SDS). The slurry was incubated with 100 µg/mL proteinase K (Sigma-Alrich, USA) at 56^o^C for two hours with occasional, gentle mixing, cooled to room temperature and extracted with equal volume of Tris-saturated phenol-chloroform (pH 8) followed by chloroform extraction. Nucleic acids were precipitated by the addition of 0.6X volume of isopropanol and pelleted at 17900 x g for 15 mins at 25^o^C, desalted by washing with 70% ethanol, air-dried, then re-dissolved in 50 µL TE buffer. RNAs were degraded with 10 µg/mL RNase A (Wako Pure Chemical Industries, Japan) at 37^o^C for 1 hour. Proteinase-K digestion, organic extraction, and isopropanol precipitation were repeated to remove the RNase and most of the degraded RNA. The resultant nucleic acid pellet was redissolved in 50 µL of 10 mM Tris-HCl, pH 8.0 and quantitated using Nanodrop 2000 (Thermo Fisher Scientific, USA). Approximately 20 ng of the extracted DNA was pre-stained with 1:600 GelRed (Biotium, USA) and eletrophoresized in 1% agarose gel, 0.5X TAE buffer for 1 hour to check for integrity.

One microgram of the genomic DNA was digested with 20 units of MspI (New England BioLabs, USA; a.k.a. NEB) in 50 µL of 1X NEB Buffer 2 at 37^o^C overnight. Two µL of the digestion product were electrophoresized in 1% agarose gel (0.5X TAE) to ensure complete digestion. The digestion product was cleaned up via phenol-chloroform extraction, ethanol precipitated and re-dissolved in 10 mM Tris-HCl, pH 8. End-filling and dA-tailing of the digestion product were carried out simultaneously: 300 ng of MspI-digested genomic fragments were incubated with 5 units of Klenow fragments (3'→5' exo-) and dNTP mix (10 mM dATP, 1 mM dCTP, and 1 mM dGTP) in 20 µL of NEB Buffer 2 at 30^o^C for 20 mins, then at 37^o^C for another 20 mins. The product was purified via phenol-chloroform extraction and ethanol precipitation and were re-dissolved in 15 µL of 10 mM Tris-HCl, pH 8. The purified product was ligated to 0.75 µM of custom-made adapters using 2000 units of T4 DNA ligase (NEB) in 20 µL of 1X NEB T4 ligation buffer at 16^o^C overnight. The adapter was prepared immediately before ligation by annealing 1 µL each of F3-02top and F3-02bottom (S2 Table) (synthesized by Thermo Fisher Scientific, USA) in 10 µL of annealing buffer (10 mM Tris-HCl, pH8, 50 mM NaCl, and 1 mM EDTA) via denaturation at 95^o^C for 2 mins and ramping down (at -0.1^o^C/s) to 25^o^C in a PCR machine. Ligation product was electrophoresized in parallel with the 20 bp DNA ladder (Takara Bio, Japan) in 3% agarose gel, 0.5X TBE buffer at 100V for 2 hours. The gel was post-stained in 3X GelGreen (Biotium) in 0.5X TBE buffer. The gel lane containing the fragments was excised for 160-340 bp dsDNA-equivalent (corresponding to 40-220 bp adapter-ligated fragments due to the Y-shaped adapter that reduced the molecules’ mobility in gel). Fragments were extracted from the excised gel and purified using Zymoclean Gel DNA Recovery Kit (Zymo Research, USA) according manufacturer’s instructions, except that 500 ng of sheared salmon sperm DNA (Thermo Fisher Scientific) were spiked into the dissolved gel prior to column loading to minimize sample loss during column washing. Purified DNA was eluted in 20 µL of 10 mM Tris-HCl, pH 8 into low-binding microfuge tube (Eppendorf, Germany).

Adapter-ligated fragments were enriched and amplified by PCR: 0.5 µL of eluted product, 200 µM dNTP, 300 nM each of F3-03F and F3-03R primers (containing non-template I-SceI restriction sites and bisulfite PCR primer sites for downstream use), and 10 units of PfuTurbo Cx polymerase in 200 µL (split into 8 tubes of 25 µL) of 1X PfuTurbo Cx reaction buffer (Agilent, USA) at 95^o^C 2 mins, 18 cycles of 95^o^C for 30 s, 65^o^C for 30s, and 72^o^C for 45 s, then followed by 72^o^C for 5 mins, finally held at 10^o^C until further processing. PCR product were pooled and purified with 1.8X volume of homemade SPRI magnetic beads (1:50-diluted carboxylated Sera-Mag Magnetic SpeedBeads in the SPRI buffer described above) (GE Healthcare, USA) and eluted in 42.5 µL of 10 mM Tris-HCl, pH 8. Two and a half microlitre of the purified product were run in 3% agarose gel, 0.5X TBE buffer to check for properly selected sizes. Negative control was processed in parallel using identical procedures, except MspI was replaced with Milli-Q water, and resulted in no amplification product.

**Method 3 Artificial methylation prior to injection**

The captured, amplified genomic fragments have two Dam sites (5’-GATC-3’) on the ligated adapters (one on each end; downstream of the BSP primer binding sites). The enriched CpG-rich fragments was tagged on the Dam sites via Dam methylation using 8 units of *dam* methyltransferase in 50 µL of 1X *dam* Methyltransferase Reaction Buffer (NEB) at 37^o^C overnight. Reaction product was ethanol precipitated, re-dissolved and re-incubated with fresh *dam* methyltransferase reaction mix overnight. Dam-methylated products were purified via phenol-chloroform extraction and ethanol precipitation, then re-dissolved in 20 µL of 10 mM Tris-HCl, pH 8. One liter of the purified product was used for photometric quantification using Nanodrop 2000 (Thermo Fisher Scientific). Nine microliters were aliquoted for injection and the rest was subjected to artificial CpG methylation as described below.

*In vitro* artificial CpG methylation of the DNA fragments was mediated by CpG methyltransferase M.SssI: 10 µL of the Dam-methylated DNA were incubated with 4 units of M.SssI methyltransferase, 640 nM of fresh S-adenosylmethionine (a.k.a. SAM) in 50 µL of 1X NEB Buffer 2 (NEB) at 37^o^C overnight. Reaction product was ethanol precipitated, re-dissolved and re-incubated overnight in the same volume of fresh M.SssI methyltransferase reaction mix, then purified via phenol-chloroform extraction and ethanol precipitation, finally re-dissolved in 10 µL of 10 mM Tris-HCl, pH 8. The purified product was quantitated using Nanodrop as above. All ethanol precipitation described above was carried out with spike-in of 1 µL of Ethachinmate (long, linear acrylamidic polymer) (Nippon Gene, Japan) as carrier to maximize DNA recovery.

**Method 4 Library preparation and next generation sequencing**

Integrated fragments were enriched from bisulfite-converted genomic DNA by PCR amplification. Two high fidelity polymerases, ExTaq (Takara Bio) and KAPA HiFi Uracil+ (Kapa Biosystems, USA) were separately used to amplified the fragments. The PCR primers were originally designed and optimized for the use in ExTaq reaction. However, KAPA HiFi Uracil+ was also included for its low amplification bias and to serve as a cross-reference to the ExTaq library. For ExTaq reaction: 2 µL of bisulfite-converted genomic DNA was used as template in 50 µL of ExTaq reaction with the primers F3-01F and F3-01R (500 nM each) at 95^o^C for 2 mins, 35 cycles of 95^o^C for 30 s, 60^o^C for 30 s, 72^o^C for 30 s, then 72^o^C for 10 mins, and finally hold at 10^o^C until further processing; For Kapa HiFi Uracil+ reaction: 2 µL of bisulfite-converted genomic DNA was used as template in 50 µL of KAPA HiFi HotStart Uracil+ ReadyMix reaction with the same primers (300 nM each) at 95^o^C for 3 mins, 35 cycles of 98^o^C for 20 s, 60^o^C for 15 s, 72^o^C for 15 s, then 72^o^C for 30 s, and finally hold at 10^o^C until further processing. PCR products were purified using 1.8X volume of homemade SPRI beads and eluted in 10 µL of 10 mM Tris-HCl, pH 8. Yield was measured via fluorometric quantification using Qubit dsDNA HS Assay Kit and Qubit 2.0 Fluorometer (Thermo Fisher Scientific). The remaining eluates (405 ng and 423 ng from embryos injected with Dam-methylated or Dam+CpG-methylated fragments, respectively) were ligated with FastGene Adapter Kit for Illumina (Nippon Genetics, Japan) using KAPA Hyper Prep Kit (Kapa Biosystems) according to manufacturer’s instructions. No post-ligation library amplification was performed. Routine quality check was carried out using High Sensitivity DNA Kit on the 2100 Bioanalyzer (Agilent). Quantitation of the adapter-ligated library was accomplished using GenNext NGS Library Quantification Kit (TOYOBO, Japan) in Mx3005P qPCR System (Agilent). Libraries were pooled in equal molar ratio, denatured and diluted to a final concentration of ca. 13 pM with 20% PhiX control spiked-in for sequencing as instructed (MiSeq Reagent Kit v2; Illumina, USA). The automated sequencing run was conducted in paired-end mode (150 bp from each ends). A cluster density of ca. 1200/mm^2^ was achieved with over 85% of Phred score > 30.

**Method 5 *In vitro* transcription for the generation of mRNA and sgRNA**

To produce mRNA for injection, the *in vitro* transcription template was amplified from the vector (using available standard sequencing primers other than those for T7 or SP6 promoter) and gel-purified using Zymoclean Gel DNA Recovery Kit (Zymo Research). *In vitro* transcription and polyA-tailing were carried out using HiScribe ARCA mRNA Kit (NEB) according to manufacturer’s instructions, except that the T7 enzyme mix was replaced by SP6 polymerase mix (Thermo Fisher Scientific) if the template was to be transcribed from the SP6 promoter. Capped, polyA-tailed synthetic mRNA was purified using RNeasy Mini Kit (Qiagen, USA).

sgRNAs for CRISPR-Cas9 were produced using EnGen sgRNA Synthesis Kit, *S. pyogenes* (NEB) following the supplied protocol. *In vitro* transcribed RNAs were purified via RNA Clean & Concentrator-5 (Zymo Research), pooled in equal molar amount, ethanol precipitated, and re-dissolved in freshly dispensed Milli-Q water (Merck Millipore).

In both cases, the purified RNAs were quantitated using Nanodrop 2000 (Thermo Fisher Scientific). Approx. 100 ng of the RNA was electrophoresized in 1% agarose gel, 0.5X TAE (i.e. under native condition) to check for integrity.
